# Divergent Evolutionary Pattern of Sugar Transporter Genes is Associated with the Difference in Sugar Accumulation between Grasses and Eudicots

**DOI:** 10.1038/srep29153

**Published:** 2016-06-30

**Authors:** Wei Wang, Hui Zhou, Baiquan Ma, Albert Owiti, Schuyler S. Korban, Yuepeng Han

**Affiliations:** 1 Key Laboratory of Plant Germplasm Enhancement and Specialty Agriculture, Wuhan Botanical Garden of the Chinese Academy of Sciences, Wuhan, 430074, China; 2 Graduate University of Chinese Academy of Sciences, 19A Yuquanlu, Beijing, 100049, China; 3 Department of Biology, University of Massachusetts Boston, Boston, MA 02184, USA; 4 Sino-African Joint Research Center, Chinese Academy of Sciences, Wuhan, 430074, China

## Abstract

Sugars play a variety of roles in plants, and their accumulation in seeds and/or surrounding pericarp tissues is distinctly different between grasses and eudicots. However, little is known about the evolutionary pattern of genes involved in sugar accumulation in these two major groups of flowering plants. Here, we compared evolutionary rates, gene duplication, and selective patterns of genes involved in sugar metabolism and transport between grasses and eudicots using six grass species and seven eudicot species as materials. Overall, sugar transporter genes exhibit divergent evolutionary patterns, whereas, sugar metabolism genes showing similar evolutionary pattern between monocots and eudicots. Sugar transporter genes have higher frequencies of recent duplication in eudicots than in grasses and their patterns of evolutionary rate are different. Evidence for divergent selection of these two groups of flowering plants is also observed in sugar transporter genes, wherein, these genes have undergone positive selection in eudicots, but not in grasses. Taken together, these findings suggest that sugar transporter genes rather than sugar metabolism genes play important roles in sugar accumulation in plants, and that divergent evolutionary patterns of sugar transporter genes are associated with the difference of sugar accumulation in storage tissues of grasses and eudicots.

Monocots and eudicots are the two major classes of angiosperms. Eudicots includes many economic crops such as soybean (*Glycine max*), castor-oil plant (*Ricinus communis*), tomato (*Solanum lycopersicum*), and fruit trees such as apple (*Malus* × *domestica*) and orange (*Citrus sinensis*). Grasses (Poaceae) are the most economically important family of monocots and contain many important cereal crops, including rice (*Oryza sativa*), foxtail millet (*Setaria italica*), and corn (*Zea mays*), among others. Monocots and eudicots show distinct differences in morphology and physiology, such as the structural features and storage compounds of seeds, although little is known about the mechanism underlying the differences^1^. In general, monocot seeds have endosperms at maturity and serving as storage tissues, whereas, mature seeds of eudicots lack endosperms, and cotyledons serve as major storage organs. Moreover, monocot seeds are usually rich in starch, whereas, eudicot seeds are rich in proteins and/or lipids. All these energy-rich storage compounds are initially derived from sugars, the primary products of photosynthesis (Fig. 1). In addition, the maternal pericarp enclosing the seed in monocots functions as a transitory storage organ during early caryopsis (grain fruit) development and the pericarp starch is remobilized into the rapidly expanding endosperm when the grains reach maximum lengths^2^. In contrast, in eudicots such as fruit trees, the pericarp serves as the main storage organ and accumulates large amounts of sugars. Therefore, sugar metabolism in seed, along with the surrounding pericarp, is significantly different between monocots and eudicots. Sugars are the major components of edible horticultural crops such as fruits and vegetables and they have a strong impact on the overall organoleptic quality of fruits and vegetables. Thus, understanding the mechanism(s) that drive sugar accumulation in plants will lead to novel ideas for increasing sugar yields, not only for fruit and vegetable crops, but also for bioenergy crops as well.

Sugars, the primary products of photosynthesis, function as energy-storage and carbon transport molecules and substrates for starch, protein, and lipid metabolism. Sugars also function as osmotica and hormone-like primary messengers in signal transduction. Therefore, sugars perform a variety of roles in plants^3^. Sugar metabolism in plants comprises a complex network of multiple pathways, and involves in cytosol and several organelles, such as vacuoles and amyloplasts (Fig. 1). In sink cells, sucrose is converted to fructose and glucose by neutral invertase (NI). Fructose and glucose are further converted to fructose-6-phosphate (F6P) and Glc-6-phosphate (G6P) by fructokinase (FK) and hexokinase (HK), respectively. Moreover, sucrose can also be converted to G6P via three successive reactions catalyzed by sucrose synthase (SUSY), UDP-glucose pyrophosphorylase (UDPase), and phosphoglucomutase (PGM), respectively. Both G1P and G6P are used for starch synthesis, whereas, F6P can be combined with UDP-glucose (UDPG) to re-synthesize sucrose via suc-phosphate synthase (SPS) and suc-phosphate phosphatase (SPP). In addition, G6P and F6P can interconvert into each other via phosphoglucose isomerase (PGI). Soluble sugars are transported into vacuole by transporter proteins located on the vacuole membrane, such as sucrose transporter (SUT) and monosaccharide transporter (MST)^4^. The MST family is diverse and contains seven distinct subfamilies, sucrose transporter (SUT), tonoplastic monosaccharide transporter (TMT), vacuolar glucose transporter (VGT), polyol/monosaccharide transporter (PMT), sugar transporter protein (STP), inositol transporter (INT), plastidic glucose transporter (pGlcT), and early-responsive to dehydration (ERD6)-like transporter^5^. Genes involved in sugar metabolism and transport between cytosol and vacuole have been identified in numerous plants, and their functions have been found remarkably conserved across all eudicots and grasses^3^,^6^.

In plants, sugar accumulation is dependent upon two processes, biosynthesis and transport. To date, many studies have been conducted to investigate regulatory mechanisms of sugar accumulation^7^,^8^,^9^. Some studies have reported that genes involved in sugar biosynthesis, including *NI*, *SUSY, HK,* and *SPS*, play important roles in sugar accumulation^10^,^11^,^12^,^13^,^14^, while other studies have argued that genes involved in sugar transport, such as SUT and MST families, are important for sugar accumulation^15^,^16^,^17^. Therefore, additional studies are needed to clarify whether it is sugar biosynthesis or transport that is more important in sugar accumulation.

More recently, several studies reveal evolutionary patterns of genes involved in starch biosynthesis in plants. For example, Yu *et al*.^18^ reported the strong evidence of positive selection at *AGPL*, *GBSSI* and *SBEII* in rice wild ancestor^18^. Li *et al*.^19^ found that divergent evolutionary pattern of genes involved in starch biosynthetic pathway between grasses and eudicots contributes to the observed differences in starch biosynthesis in seeds^19^. However, there are few reports on the mechanisms underlying the observed sugar accumulation difference in seeds and their surrounding pericarps between grasses and eudicots. In this study, we compared the evolutionary rates, gene duplication, and selective patterns of genes involved in sugar accumulation between grasses and eudicot plants, including four above-mentioned key gene families involved in sugar metabolism, *HK*, *NI*, *SPS*, and *SUSY,* and two sugar transporter gene families, *SUT* and *MST*. It is worth noting that a newly identified sugar transporter family called SWEET is involved in sugar transport in plants^20^. However, most SWEET genes play an important roles in phloem transport and are related to biotic and abiotic stress^21^,^22^,^23^,^24^,^25^. Since phloem transport is not a limited step for sugar accumulation in storage organs such as seeds or fruits^26^, the SWEET family was not included in this study. Our aim is to determine whether genes involved in either sugar metabolism or transport are most critical for sugar accumulation in plants. Our study not only provides insights into the observed divergent evolutionary patterns of genes involved in sugar accumulation in plants, but will also contribute to understanding of the observed morphological and physiological differences between grass and eudicot plants.

## Materials and Methods

### Retrieval and Comparison of Sequences

Amino acid sequences of genes involved in sugar accumulation and transport were collected using the protocol described by Tatusov *et al*.^27^, but with slight modifications. Briefly, the main steps were as follows: (1) Protein-coding transcripts of *Brachypodium distachyon* (v2.1 release)^28^, *Oryza sativa* (MSU Release 7.0)^29^, *Setaria italic* (v2.1 release)^30^, *Sorghum bicolor* (v2.1 release)^31^, *Zea mays* (6a release)^32^, *Arabidopsis thaliana* (TAIR release)^33^, *Citrus sinensis* (v1.1 release)^34^, *Malus* × *domestica* (v1.0 release)^35^, *Glycine max* (Glyma1.0, Soybean Genome Project)^36^, *Ricinus communis* (release v0.1)^37^, *Solanum lycopersicum* (iTAG2.3 release)^38^, *Panicum virgatum* (v1.1), and *Populus trichocarpa* (v3.0)^39^ were downloaded from either JGI (http://www.phytozome.net/) or the Rice Genome Annotation Project (http://rice.plantbiology.msu.edu/) and a local BLAST database was constructed for each species using BLAST 2.2.24; (2) An all-against-all protein sequence comparison was carried out to identify homologous genes of *Arabidopsis* or *Oryza sativa* in eudicots and grasses, respectively; (3) In all the comparisons performed in the step 2, the orthologs were further identified using BLAST 2.2.24 with *SUT2* in *Arabidopsis* or rice as a query, and obvious paralogs were collapsed; (4) All interspecies Best Hits (BeTs) of *AtSUT2* or *OsSUT2* and their paralogs were detected; (5) Steps 3 and 4 were repeated, with other *SUT* genes rather than *SUT2* as BLASTp queries until all the *SUT* genes were identified; (6) Clusters of Orthologous Groups (COGs) were formed for the *SUT* gene family using the protocol previously reported by Tatusov *et al*.^27^; (7) COGs were constructed for other gene families using the same protocol as conducted for the *SUT* gene family.

### Estimation of Gene Duplication and Gene Loss

Within each gene family, gene duplication and loss were analyzed by checking of each subfamily manually. Each gene family was assumed to have at least two subclades, grass and eudicot subfamilies, with at least six and seven members in grass and eudicot subfamilies, respectively. If there are two or more grass or dicot subclades, or if a subclade has two or more members from the same species, it was assumed to have one or more duplications. If a member was found to be missing from any species within the grass or eudicot subclade, it was concluded that there was a gene loss. Each gene loss was further validated by searching GenBank database using BLASTp.

### Phylogenetic Analyses

Amino acid sequences were aligned using CLUSTAL X and manually adjusted using Seaview (Galtier *et al*.^40^. The resulting data matrix was used to construct a phylogenetic tree using the MEGA software (v6.06)^41^ with both Neighbor Joining (NJ) and Maximum Likelihood (ML) methods. For the NJ method, the parameters were as follows: model, p-distance; bootstrap, 1,000 replicates; and gaps/missing data treatment, pairwise deletion. For the ML method, the parameter setups were as follows: model, Jones-Taylor-Thornton (JTT); bootstrap, 1,000 replicates; gaps/missing data treatment, partial deletion; and branch swap filter, very strong.

### Estimation of d_N_:d_S_ Ratio

The codon sequences were aligned using MEGA v7.0 software with muscle codons alignment model, and termination codons were manually deleted. The alignment result was used for estimation of the ω values using maximum likelihood method in Codeml from the PAML v4.9a^42^. The significance of variation in the ω value among different branches in gene trees was tested using Codeml, a branch-specific model. This branch-specific model can be compared with one-ratio model that assumes a constant ω value across all branches using the likelihood ratio test (LRT).

Coding DNA sequences were aligned using CLUSTAL X and adjusted manually, as deemed necessary. The resulting data matrix was used to estimate ω value, the ratio of nonsynonymous substitutions per nonsynonymous site (d_N_) to synonymous substitutions per synonymous site (d_S_) of homologous gene pairs. The estimation of ω value was performed with the maximum likelihood method using a KaKs_Calculator package (v1.2)^43^,^44^.

The variation of the ω ratio among different branches in gene trees was tested using the pairwise comparison approach^19^. Briefly, the ω ratios between two randomly selected sequences within the grass or eudicot subclade were calculated using the KaKs_Calculator package. The difference of the ω ratios were compared between grass and eudicot subclades with the paired *t*-test using SPSS, and the comparison was only conducted between orthologous subclades. Significant difference was set as *P* < 0.05.

### Identification of Positively Selected Sites

The codon sequences from grasses or eudicots were aligned using the online program MAFFT (http://mafft.cbrc.jp/alignment/server/), and the results were saved in PHYLIP format files. The aligned sequences were used to construct phylogenetic trees using the MEGA software (v6.06)^41^ with Neighbor Joining (NJ), and the results were stored in tree files. The PHYLIP format files, along with the trees file, were used for estimating d_N_:d_S_ ratios with maximum likelihood method using the program Fitmodel (version: 0.5.3)^45^. The parameter setups were as follows: data type, DNA; DNA substitution model applies, codons; genetic code, universal; model of codon substitution, M3; model of swtiches between selection reigns, S1 or not; start with default values for switching rates, yes; start with default dn/ds ration parameters, yes; Ts/tv ratio, fixed; codon frequency, F3 × 4; analyze multiple data sets, no; optimis substitution parameters, yes. The d_N_:d_S_ ratios were subsequently used to estimate positively selected sites in grasses or eudicots. Moreover, switching parameters were also estimated using the models M3 and M3 + 1, which were described by Yang and Nielsen^46^ and Guindon *et al*.^45^.

### Detection of Functional Divergence after Gene Duplication

Estimation of gene functional divergence after gene duplication was conducted using the Type II model in DIVERGE v3.0 software^47^. The amino acid sequences from grasses and eudicots were aligned using MEGA v7.0 and the results were saved as FASTA format. NJ phylogenetic tree was constructed using PHYLIP v3.6 software following the instructions of the DIVERGE v3.0 software. In addition, each gene subfamily within the same phylogenetic tree was treated as one cluster.

## Results

### Phylogenetic Analyses

**Figure 2:**
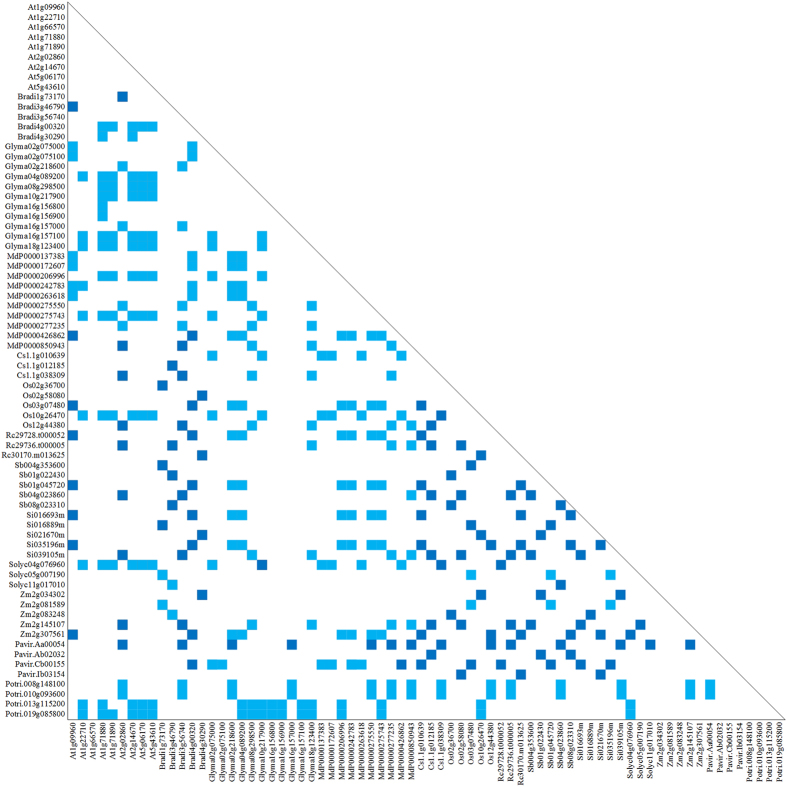
Clusters of orthologous groups (COG) of the *SUT* gene family in plants. Deep and light blue squares correspond to symmetrical and asymmetrical best hits (BeTs), respectively. Each gene ID is indicated, and the prefix “Rc” denotes IDs from *Ricimus communis*.

**Figure 3:**
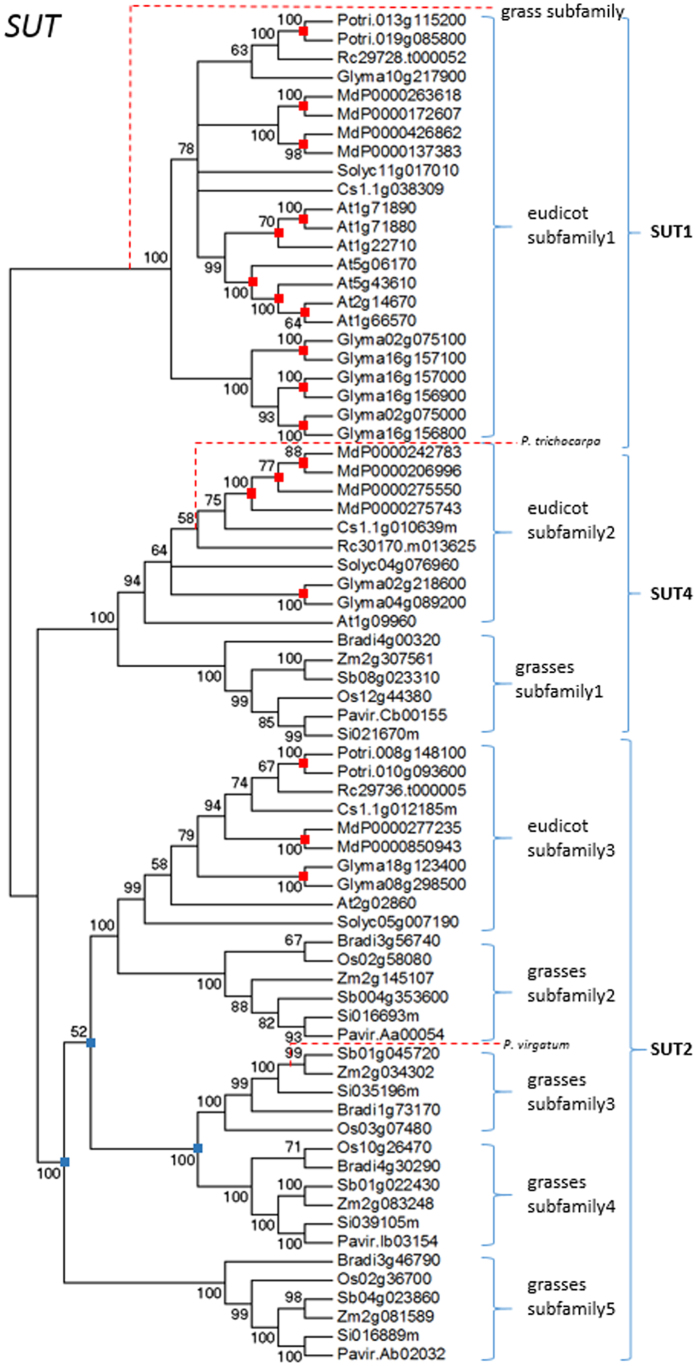
Analysis of phyogenetic relationships between SUT proteins in plants using the Neighbor-Joining method. Bootstrap values (higher than 50%) are shown near branched lines. Recent and old duplication events are indicated by red and blue dots, respectively. Gene loss is indicated by red dash line. Genes are designated based on previous reports or BLAST results.

Twelve COGs were obtained and are shown in Fig. 2 and supplementary Fig. S1. Homologs of twelve gene families, *HK*, *NI*, *SPS*, *SUSY*, *SUT*, *TMT*, *VGT*, *PMT*, *STP*, *ERD6-like*, *pGlcT*, and *INT*, were included in a single COG (Fig. 2 and supplementary Fig. S1). Phylogenetic trees showed that both NJ and ML methods yielded identical topologies (Fig. 3 and supplementary Fig. S2). Each gene family consisted of multiple subfamily clades, which were supported by high levels of confidence (mostly > 90% bootstrap value) in all phylogenetic trees. For example, the *SUT* gene family was composed of three eudicot subfamilies and five grass subfamilies (Fig. 3), and all these eight subfamilies were clustered into three clades (*SUT1*, *SUT2*, and *SUT4*). This is consistent with earlier finding that the *SUT* gene family in *A. thaliana* is composed of three types^48^. However, all *SUT* genes in grasses were grouped into the *SUT2* and *SUT4* types, with none belonging to the *SUT1* type (Fig. 3). As for the *SUT1* type, gene duplication occurred frequently in most eudicot species such as *A. thaliana* following the monocot-eudicot split. For the *SUT2* type, gene duplication occurred twice in grasses during the process of speciation. In addition, both grasses and eudicots contain *SUT2* and *SUT4* types, which suggested that the duplication of *SUT* genes have also occurred prior to the monocot-eudicot split. In short, gene duplication events in the *SUT* gene family have occurred at various time points during the process of plant evolution, covering both before and after the monocot-eudicot split.

Similar to the *SUT* gene family, the *MST* gene families, *TMT*, *VGT*, *PMT*, *STP*, *ERD6-like*, *pGlcT*, and *INT*, are also composed of multiple subfamilies (supplementary Fig. S2). The *STP* gene family contained the most abundant subfamilies, including nine grass subfamilies and eight eudicot subfamilies, while the *VGT* gene family had the lowest number of subfamilies, including two grass subfamilies and two eudicot subfamilies. The rest five transporter gene families, *TMT*, *PMT*, *ERD6-like*, *pGlcT*, and *INT*, contained 3–5 grass subfamilies and 3–4 eudicot subfamilies. Most duplication events of the *MST* gene families have primarily occurred following the monocot-eudicot split. Gene loss event was observed in all the *MST* gene families except the *VGT* family.

For sugar metabolism gene families, both *HK* and *NI* families contained eight grass subfamilies and four eudicot subfamilies, while *SPS* and *SUSY* families consisted of 5–6 grass subfamilies and 4–5 eudicot subfamilies. Similar to *SUT* genes, duplication events of these sugar metabolism genes have occurred both before and after the monocot-eudicot split.

### Differences in Gene Duplication and Loss Patterns between Grasses and Eudicots

It has been reported that gene duplication can be classified into either recent or old duplications according to the time of occurrence^49^. In this study, both grass-specific (duplication prior to radiation of grasses) and eudicot-specific (duplication prior to radiation of eudicots) duplications were designated as old duplications, whereas, recent duplications were deemed as species-specific duplications (duplication within a species). Both recent and old duplications were investigated for each gene family (Table 1). In grasses, old duplication occurred approximately 26 times in ten gene families (*PMT, HK, SUT, SUSY, PMT, TMT, NI*, *SPS, ERD6-like,* and *INT*), but only five times in five gene families (*SPS, HK, SUSY, STP* and *TMT*) in eudicots. In addition, recent duplication in grasses occurred 17 times in six gene families (*NI, SPS, HK, TMT, STP*, and *SUSY*), whereas, recent duplication occurred 144 times in all the tested gene families in eudicots. The *STP* genes showed the highest frequency of recent duplication in eudicots (34 times). In short, old duplication frequently occurred in grasses, while recent duplication frequently occurred in eudicots. In addition, it is interesting to note that the frequencies of recent duplications showed significant variations among tested eudicot species, with most recent duplications observed in the following two species, *M.* ×*domestica* and *G. max*.

Recent gene loss in grasses was observed in ten gene families, including *HK*, *TMT*, *NI*, *STP*, *SPS*, *PMT*, *SUT*, *pGlcT*, *ERD6-like,* and *INT* (Table 1). Similarly, recent gene loss in eudicots was also observed in ten gene families, including *HK*, *TMT*, *NI*, *STP*, *SPS*, *PMT*, *SUT*, *pGlcT*, *ERD6-like,* and *SPS*. Moreover, old gene loss was only observed in the *SUT1* clade in grasses (Table 1, Fig. 3). Overall, recent gene loss events occurred frequently in both grasses and eudicots, while old gene loss event rarely occurred in both grassed and eudicots (Table 1, Fig. 3, and supplementary Fig. S2).

### Most Genes Involved in Sugar Metabolism and Transport Have Rapidly Evolved in Grasses

**Figure 4:**
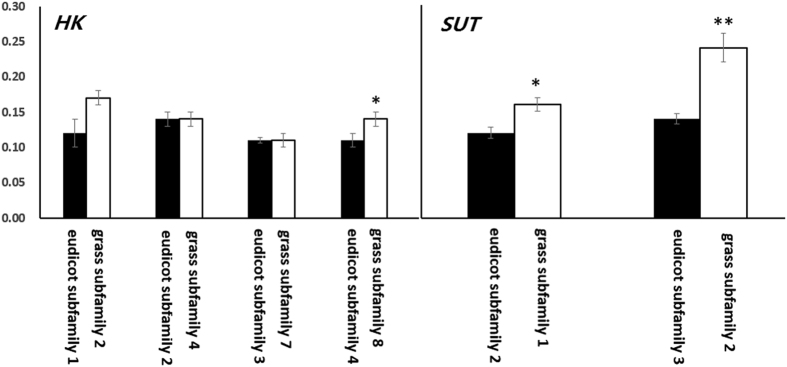
Differences in ω values of *SUT* and *HK* gene subfamilies between grasses and eudicots. Black and white columns correspond to ω values of gene subfamilies in eudicots and grasses, respectively. Error bars correspond to SE of means. *P < 0.05; **P < 0.01.

To determine whether sugar metabolism and transport gene families are under different evolutionary constraints in both grasses and eudicots, pairwise ω (d_N_/d_S_) values for all tested genes were calculated using the pairwise comparison model. The pairwise ω values within each subfamily were subsequently compared between grasses and eudicots. As a result, 10 subfamilies within the *SUT, HK, VGT, PMT, STP* and *pGlcT* gene families showed significantly higher mean ω values in grasses than in eudicots (Fig. 4, Supplementary Table S1), whereas, 13 subfamilies within the *STP*, *TMT*, *INT*, *pGlcT,* and *ERD6-like* families showed significantly higher mean ω values in eudicots than in grasses (Supplementary Table S1). This finding suggests that the majority of the genes involved in sugar transport are significantly different in evolutionary rates between grasses and eudicots.

Since pairwise comparison approach model cannot calculate the evolutionary rate of the most recent common ancestor of the subfamily, the branch-specific model was selected to check whether there are the changes in the evolutionary constraints across different subfamilies. As a result, all the metabolic gene families had *p*-values larger than 0.05, whilst almost all sugar transport gene families except *VGT* had *p*-values smaller than 0.05 (Table 2). This indicates that the evolutionary rate of sugar transport gene families is different between grasses and eudicots.

**Figure 5:**
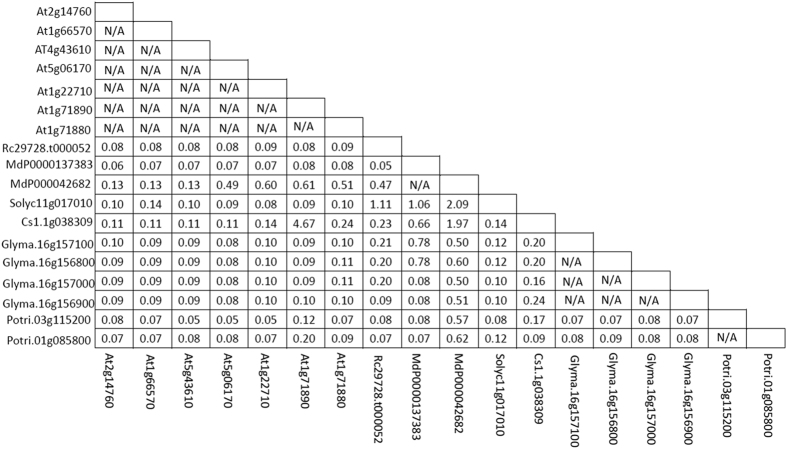
Estimation of ω values for the *SUT1* subfamily using the ML method. Values higher than one are highlighted in black bold.

In addition, we compared the ω values between members within the *SUT1* clade. Interestingly, all the ω values in three fruit crops, *S. lycopersicum*, *C*. *sinensis*, *Malus* × *domestica*, were higher than 1.0 (Fig. 5). This suggests that *SUT1* in these three fruit crops has higher evolutionary rates compared with other eudicots investigated in this study.

### A Divergent Selection of Sugar Transporter Gene Families Observed in Grasses versus Eudicots

To assess the potential for selection of genes involved in sugar metabolism and transport, the Fitmodel software was used to conduct selection analysis with both M3 and M3 + S1 models^45^, and the sites under positive selection for each gene family were compared between grasses and eudicots. Parameter estimates for selection analysis were found to be similar between M3 and M3 + S1 models (Table 3), thus, only results from M3 + S1 model analysis was presented.

Overall, more than 85% of sites (p1 and p2) in all tested genes, except for *NI*, were identified to be under purifying selection with ω1 ≤ 0.18 and ω2 ≤ 0.87. Moreover, 1% to 15% of identified sites (p3) within ten sugar transporter genes (*SUT1*, *SUT2*, *SUT4*, *STP*, *VGT*, *TMT*, *PMT, pGlcT*, *ERD6-like,* and *INT*) and one sugar metabolism gene *NI* were identified to be under positive selection with an ω3 value > 1.0. Among these positive selection sites, the number of sites for genes in *SUT1*, *SUT2*, *TMT*, *PMT, VGT, pGlcT*, *ERD6-like,* and *INT* gene families were significantly asymmetric across both grasses and eudicots (Table 4). This suggests that selection is mainly located either in the grass or eudicot subclade of the phylogenetic tree, thus providing a key evidence of the incidence of divergent selection between grasses and eudicots.

### Type II Functional Divergence after Gene Duplication

The coefficient of type II functional divergence (θ) between any two clusters was calculated using DIVERGE v3.0. For sugar transport gene families, non-orthologous clusters had θ-II values that were significantly larger than 0, but not for orthologous clusters (Table 5, Supplementary Table S2). However, such a pattern was not observed between any two clusters of sugar metabolic gene families (Supplementary Table S2). This suggests that type II functional divergence may occur between sugar transport gene subfamilies.

## Discussion

In this study, evolutionary features such as duplication patterns, evolutionary rates, and positive selection have been investigated for twelve gene families involved in sugar metabolism and transport in plants, including *HK*, *NI*, *SPS*, *SUSY*, *PMT*, *STP*, *SUT*, *TMT, VGT, pGlcT*, *ERD6-like,* and *INT*. Overall, sugar transporter gene families have demonstrated divergent evolutionary pattern between grasses and eudicots, whereas, sugar metabolism gene families have revealed similar evolutionary patterns between grasses and eudicots. For example, the *SUT1* clade is found to be exclusively present in eudicots, and *SUT2* genes show high frequencies of old gene duplication in grasses than in eudicots. Moreover, *STP* genes exhibit higher frequency of recent duplication in eudicots than in grasses, whereas, they show higher frequency of old duplication in grasses than in eudicots. In addition, both *SUT2* and *SUT4* gene families in grasses have faster evolutionary rates than in eudicots. Given the fact that sugar accumulation in seeds and/or pericarps is significantly different between grasses and eudicots, we hypothesize that this observed difference is associated with divergent evolutionary patterns of sugar transporter gene families.

### Hypothesis 1 - Gene Duplication of Sugar Transporters Contributes to Sugar Accumulation in Eudicots

In this study, almost all analyzed transporter genes in eudicots, including *PMT, STP, SUT*, *TMT*, *VGT, pGlcT*, *ERD6-like,* and *INT*, have undergone one or more rounds of duplication in eudicots (Table 1, Fig. 3, and supplementary Fig. S2). The *SUT* genes serve as a typical example of this finding as they have undergone duplication eighteen times in eudicots.

In plants, the *SUT* gene family encodes sucrose/H^+^ symporters, and it consists of three types, termed *SUT1*, *SUT2* and *SUT4*^50^,^51^,^52^,^53^. In this study, the *SUT1* subfamily is found to be present exclusively in eudicots. Previous studies show that both SUT1 and SUT2 proteins are localized to plasma membrane^54^,^55^, while SUT4 protein is localized to vacuolar membrane^56^,^57^. The *SUT1* genes are expressed in both source and sink tissues and play essential functions such as those of phloem loading and increasing sucrose uptake into sink storage tissues^58^,^59^,^60^,^61^,^62^. On the other hand, *SUT2* genes are mainly expressed in sink tissues and function in phloem loading^63^,^64^,^65^,^66^. Whereas, *SUT4* genes show variable patterns of expression in different plant species and function in sucrose uptake from the vacuole into the cytoplasm^61^,^62^,^63^,^64^,^65^,^66^,^67^,^68^,^69^. In higher plants, it is commonly known that sucrose is the main mobile carbohydrate. *SUT1* genes exhibit very high affinity and transport ability with sucrose^70^,^71^,^72^,^73^, while *SUT2* and *SUT4* genes have no or low affinity for sucrose^55^,^74^,^75^,^76^,^77^. Moreover, both *SUT2* and *SUT4* genes are not reported to increase sucrose uptake into the sink storage tissues. Thus, the *SUT1* subfamily is deemed critical for sugar transport and its duplication is likely to be associated with observed high levels of sugar accumulation in eudicots. In turn, loss of the *SUT1* subfamily in grasses is probably one of the main causes leading to observed differences of sugar accumulation in storage tissues between grasses and eudicots.

In this study, it is found that *SUT2* genes, unlike *SUT1* genes, show high frequencies of old duplication in grasses, but no duplication detected in eudicots. *SUT2* genes are reported as potential candidates for starch accumulation^55^. Thus, duplication of *SUT2* genes may contribute to starch accumulation in grasses.

Besides the *SUT* gene family, the *STP* gene family also functions as sugar transporters^69^,^78^,^79^,^80^. The *STP* gene family, also known as hexose transporters (HTs), is located in the plasma membrane, functions as monosaccharide/proton symporter, and plays an important role in monosaccharide import into sink cells^80^. In fruits, several studies have demonstrated that the *STP* gene family plays important roles in sugar accumulation^81^,^82^,^83^. In this study, the *STP* gene family is found to show differences in duplication patterns between eudicots and grasses. In grasses, the *STP* gene family has undergone four duplications in ancient times, but six duplications in recent times. In eudicots, the *STP* gene family has undergone only one duplication in ancient times, but has also undergone 34 recent duplications as well. Interestingly, the duplication of the *STP* gene family in recent times has reached as high as 24 times in three fruit crops, apple, tomato, and orange. This suggests that gene duplication of the *STP* subfamily may also contribute to sugar accumulation in eudicots. In addition, the rest six transporter gene families, *PMT, TMT*, *VGT, pGlcT*, *ERD6-like,* and *INT*, show similar duplication patters to *SUT* and *STP* gene families, which suggests that they are may also play important roles in sugar accumulation. However, sugar metabolism genes, unlike sugar transporter genes, show no significant differences in gene duplication patterns between grasses and eudicots (Fig. S2), thus indicating that they are unlikely responsible for observed differences in sugar accumulation between eudicots and grasses.

Taken together, all these findings support our hypothesis that sugar transporter gene duplication plays an important role in sugar accumulation in eudicots. In addition, our study reveals that functional divergence may occur after gene duplication between sugar transport gene subfamilies. More experiments are needed to clarify the roles of sugar transport gene subfamilies in sugar accumulation.

### Hypothesis 2 - Evolutionary Rate Patterns of Sugar Transporters are Associated with Differences in Sugar Accumulation between Eudicots and Grasses

The ω value is an important index of genetic differentiation. In this study, both one-ratio model and a branch model were conducted to estimate the ω value, and the likelihood of these two models was compared using LRT statistic. The result reveals that evolutionary rates of most sugar transporter gene families are significantly different between grasses and eudicots. In contrast, no significant difference is observed for most sugar metabolism gene families between grasses and eudicots (Table 2). Among all tested genes in this study, those with ω > 1 are exclusively found in the *SUT1* subfamily. Interestingly, all *SUT1* genes with ω > 1 are derived from fruit crops, including apple, orange, and tomato (Fig. 5). This indicates that *SUT1* genes in fruit crops have faster evolutionary rates than those in other eudicot species. As fruits are rich in sugars, we hypothesize that faster evolutionary rates of *SUT1* genes contribute to sugar accumulation in eudicots.

In grasses, the *SUT2* clade consist of four subfamilies. In addition, grass subfamily 2 has an orthologous relationship with the eudicot subfamily 3. The ω value for grass subfamily 2 is significantly higher than that for the eudicot subfamily 3. Similarly, *SUT4* genes have significantly higher ω values in grasses than those found in eudicots. These results suggest that both *SUT2* and *SUT4* genes have significantly different evolutionary rates between grasses and eudicots. Several studies have reported that SUT2 is a sucrose sensor that can interact with SUT1 and/or SUT4 to regulate their relative activities^55^,^75^,^84^. Thus, the evolutionary rates of both *SUT2* and *SUT4* genes are also likely associated with sugar accumulation in plants.

As with *SUT* genes, other sugar transporter genes, such as *STP, VGT, PMT, TMT*, *pGlcT*, *ERD6-like,* and *INT*, have shown significant differences in ω values between grasses and eudicots. In a recent study, it is reported that rapid evolution of starch pathway genes contributes to starch accumulation in grasses^20^. Therefore, it is most likely that the evolutionary rate of sugar transporter gene families has influences on sugar accumulation in plants.

Unlike sugar transporter gene families, almost all gene families involved in sugar metabolism, except for *HK4*, show no significant differences in ω values between grasses and eudicots. The *HK* gene is responsible for hexose phosphorylation, and plays a vital role in plant sugar sense and sugar signal transduction^85^,^86^,^87^. The HK protein is localized in different organelles, including cell cytosol, chloroplastid, chondriosome, plasmid, and golgiosome^88^,^89^. In an earlier study, it has been demonstrated that the *HK4* gene is incapable of catalyzing phosphorylation of hexoses, such as glucose or fructose^87^. Thus, it is clear that the patterns of evolutionary rates of *HK* genes involved in hexose phosphorylation are not associated with observed differences in sugar accumulation between grasses and eudicots. Such a phenomenon is quite commonly observed in genes involved in sugar metabolism (supplementary Table S1).

In this study, positive selection analysis has revealed that sites with ω3 values higher than one have been identified for all analyzed transport genes. However, positive selection sites are present exclusively in sugar transporter gene families in eudicots. This finding also demonstrates that sugar transporter genes are likely associated with high levels of sugar accumulation in eudicots.

In conclusion, sugar transporter genes rather than sugar metabolism genes play important roles in sugar accumulation in plants, and differences in sugar accumulation in storage tissues between grasses and eudicots may be attributed to at least three reasons as follows: 1) sugar transporter genes have higher frequencies of recent duplications in eudicots rather than in grasses; 2) patterns of evolutionary rates of sugar transporter genes are different between grasses and eudicots; and 3) sugar transporter genes have undergone positive selection in eudicots, but not in grasses. In short, divergent evolutionary patterns of sugar transporter genes are responsible for differences in sugar accumulation between grasses and eudicots. However, molecular mechanisms underlying the evolutionary divergence of sugar transporter genes between grasses and eudicots remain unclear.

As mentioned above, the *SUT* gene family highly influences sugar accumulation in plants, especially in fruit crops. During fruit development, sucrose is imported into the fruit, and it is subsequently cleaved into glucose and fructose. These latter sugar components not only contribute to fruit sweetness, but they can also be used as sources of energy for fruit growth. By increasing the uptake of sucrose into developing fruits, this may contribute to increased levels of sugar content in mature fruits. Therefore, manipulation of *SUT* genes may lead to enhanced sugar content in fruit crops.

## Additional Information

**How to cite this article**: Wang, W. *et al*. Divergent Evolutionary Pattern of Sugar Transporter Genes is Associated with the Difference in Sugar Accumulation between Grasses and Eudicots. *Sci. Rep.* **6**, 29153; doi: 10.1038/srep29153 (2016).

## Supplementary Material

Supplementary Information

## Figures and Tables

**Figure 1:**
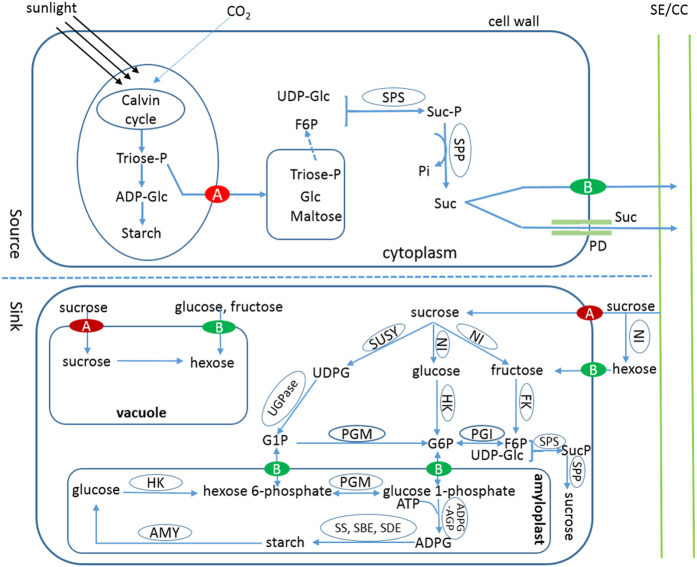
Sucrose (Suc) synthesis, metabolism and transport in plant cell. ADP-Glc: ADP-glucose; Glc: glucose; triose-P: triose-phosphate; G6P: Glc-6-phosphate; G1P: Glc-1-phosphate; F6P: fructose-6-phosphate; UDPG: uridine diphosphate glucose; SE/CC: sieve element/companion cell; Suc-P: Suc-phosphate; SPS: Suc-phosphate synthase; SPP: Suc-phosphate phosphatase; PGI: phosphoglucose isomerase; NI: neutral invertase; SUSY: sucrose synthase; HK: hexokinase; FK: fructokinase; PGM: phosphoglucomutase; UDPase: UDP-glucose pyrophosphorylase; SS: starch synthase; SBE: starch branching enzyme; SDE: starch debranching enzyme; AMY: amylase; A: sucrose transporter; and B: sugar transport protein.

**Table 1:** Gene duplication and gene loss in gene families involved in sugar metabolism and transport in both grasses and dicots.

Species	Duplication type	Genes
Grass-specific	Old gene duplication	*SPS*(2)*, HK*(5)*, NI*(3)*, SUSY*(3)*, PMT(1), STP(4), SUT(3), TMT(2), ERD6-like(2), INT(1)*
	Recent gene duplication	*SPS*(3)*, HK*(4)*, NI*(3)*, SUSY*(3)*, TMT(2), STP(2)*
	Old gene loss	*SUT*
	Recent gene loss	*HK, NI, SUSY, STP, TMT, ERD6-like,INT, pGlcT, SUT, PMT*
Eudicot-specific	Old gene duplication	*SPS*(1)*, HK*(1)*, SUSY*(1)*, STP(1), TMT(1),*
	Recent gene duplication	*SPS*(5)*, HK*(10)*, NI*(7)*, SUSY(9), PMT(15), STP(34), SUT*(18)*, TMT(9), VGT(4), ERD6-like*(16), *INT*(12), *pGlcT*(5)
	Old gene loss	N/D
	Recent gene loss	*HK, SUSY, PMT, STP SUT*, *NI, SPS, ERD6-like, pGlcT, TMT*

**Table 2:** Likelihood-ratio test (LRT) statistic and parameters from Branch Model of PAML.

Gene family/subfamily	Null hypothesis		Alternative hypothesis			LRT	
	-In L	ω	-In L	ω (grass)	ω (edicot)	Statistic	*P*
HK	11783.30	0.10	11785.01	0.10	0.13	3.42	0.18
NI	16479.90	0.06	16479.89	0.06	0.07	0.01	1.00
SPS	19549.54	0.10	19549.53	0.10	0.14	0.01	1.00
SUSY	23700.03	0.09	23700.04	0.08	0.09	0.01	1.00
SUT type1	10101.69	0.12	100094.07	N/A	0.14	15.23	4.93e–4
SUT type2	5594.01	0.29	5599.79	0.14	0.48	11.55	3.10e–3
SUT type4	4056.79	0.35	4107.06	0.10	0.76	100.54	<1.00e–12
PMT	13163.31	0.11	13172.08	0.14	0.07	17.55	1.54e–4
STP	13959.40	0.07	13903.48	0.11	0.05	111.84	<1.00e–12
ERD6-like	10500.44	0.56	10614.28	0.33	0.14	227.67	<1.00e–12
pGlcT	10476.77	0.11	10480.65	0.14	0.11	7.76	2.07e–2
INT	7717.63	0.08	7722.22	0.10	0.03	9.18	1.02e–2
TMT	11508.87	0.11	11308.87	0.11	0.16	400.00	<1.00e–12
VGT	9974.43	0.14	9797.18	0.13	0.15	6.01	0.05

**Table 3:** Estimation of likelihood ratios of gene families related to sugar metabolism and transport using the M3 and M3 + S1 Models in Fitmodel.

		Gene family/subfamily													
Model Parameter		*SUT1*	*SUT2*	*SUT4*	*VGT*	*STP*	*TMT*	*PMT*	*INT*	*pGlcT*	*ERD6-like*	*HK*	*NI*	*SUSY*	*SPS*
M3	-In L	14922.22	24210.88	7853.05	23354.78	15805.81	61719.92	51067.07	31257.70	19149.30	16142.70	63731.74	66066.34	73873.57	84870.00
	p1	0.38	0.21	0.20	0.37	0.46	0.15	0.17	0.03	0.06	0.44	0.32	0.08	0.15	0.14
	p2	0.39	0.49	0.60	0.35	0.35	0.50	0.48	0.63	0.67	0.39	0.35	0.47	0.37	0.46
	p3	0.23	0.30	0.21	0.28	0.18	0.35	0.35	0.34	0.27	0.17	0.33	0.46	0.48	0.40
	ω1	0.02	0.14	0.14	0.02	0.01	0.12	0.07	0.23	0.35	0.01	0.22	0.31	0.06	0.06
	ω2	0.27	0.77	2.74	0.24	0.14	0.83	0.47	1.97	1.98	0.15	0.16	1.41	0.50	0.52
	ω3	0.95	2.14	20.00	0.95	1.57	1.77	1.22	4.38	5.28	0.56	0.42	3.38	0.70	0.79
M3 + S1	-In L	14891.35	24166.60	7857.14	23250.22	15728.80	61624.07	50952.59	31239.20	19145.40	16035.70	63271.47	65900.66	73744.10	84666.08
	p1	0.44	0.20	0.20	0.50	0.58	0.18	0.22	0.05	0.06	0.60	0.41	0.09	0.20	0.20
	p2	0.34	0.50	0.60	0.35	0.31	0.53	0.52	0.67	0.67	0.36	0.34	0.52	0.35	0.48
	p3	0.23	0.30	0.20	0.15	0.10	0.28	0.26	0.28	0.27	0.29	0.25	0.39	0.45	0.32
	ω1	0.00	0.02	0.12	0.01	0.00	0.03	0.03	0.12	0.18	0.00	0.00	0.06	0.03	0.01
	ω2	0.28	0.72	2.76	0.39	0.24	0.87	0.53	2.03	1.94	0.02	0.16	1.35	0.51	0.55
	ω3	1.13	2.32	20.00	1.78	1.80	2.08	1.61	5.40	5.66	2.02	0.64	4.14	0.95	0.98

**Table 4:** Estimation of positive sites in gene families related to sugar metabolism and transport using the M3 + S1 Model in Fitmodel.

Gene family	Grass-specific	Eudicot-specific
*SUT1*	N/A	27, 245, 260, 266, 346, 403, 409, 411, 414, 425
*SUT2*	N/A	97, 211, 356, 411, 477, 541
*SUT4*	N/A	N/A
*VGT*	N/A	N/A
*STP*	N/A	N/A
*TMT*	N/A	272, 552, 841, 864
*PMT*	N/A	36, 38, 82, 87, 92, 504, 522, 632
*HK*	N/A	20, 29, 34, 61, 67, 70, 74, 83, 89, 179, 180, 181
*NI*	67, 70, 71, 92, 128, 129, 131, 134, 149, 155, 156, 163, 180, 182, 183, 192, 193, 194, 195, 203, 223, 348, 736	109, 111, 112, 124, 137, 145, 153, 156, 157, 168–171, 180, 181, 183, 190, 200, 203, 218, 219, 221, 222, 226, 231, 254–257, 262, 263, 266, 271, 277–279, 681, 718
*SUSY*	N/A	120, 135, 1455, 1492, 1674, 1677, 1678, 1679
*SPS*	82, 110–112, 115, 133, 266, 449, 514, 528, 552, 669, 798, 805, 881, 932, 995, 1003, 1049, 1061, 1064, 1068, 1070, 1088, 1092, 1094, 1099, 1102, 1104, 1129, 1132, 1142, 1148, 1180, 1181, 1182, 1229, 1232, 1235, 1238, 1242, 1251, 1254, 1256, 1282, 1303, 1304, 1305, 1310, 1329, 1324	22, 37, 41, 42, 68, 73, 90, 104, 121, 135, 136, 139, 140, 167, 170, 176–178, 181–183, 297, 313, 338, 362, 398, 429, 463, 467, 491, 505, 535, 541, 552, 554, 557, 560, 585, 601, 638, 809, 814, 827, 833, 841, 851, 869, 873, 874, 878, 891, 892, 899–901, 904, 905, 912, 914, 915, 927, 928, 929, 937, 938, 947, 951, 955, 959, 962, 964, 967, 968, 972, 976, 978, 983, 985–987, 998, 1009, 1011, 1023, 1024, 1046, 1047, 1058–1060, 1064, 1069, 1071, 1073, 1089, 1091, 1096, 1150, 1176, 1178, 1199, 1205–1208, 1210–1214, 1217, 1227, 1231
*INT*	N/A	25, 78–82, 123, 124, 137, 148, 157, 172–181, 225, 361, 463, 491, 535–542
*pGlcT*	N/A	68, 87, 94, 99, 103, 116, 129, 130, 482, 576, 608
*ERD6-like*	N/A	29, 38, 61, 74, 89, 137.233, 231, 429

**Table 5:** Estimation of type II functional divergence (θ) using the Type II of DIVERGE software.

Gene	Subfamily I	Subfamily II	θ-II value
*SUT*	Eudicot subfamily 1	Eudicot subfamily 2	0.32 ± 0.15
		Eudicot subfamily 3	0.31 ± 0.14
		Grass subfamily 1	0.21 ± 0.15
		Grass subfamily 2	0.22 ± 0.15
		Grass subfamily 3	0.36 ± 0.14
		Grass subfamily 4	0.35 ± 0.14
		Grass subfamily 5	0.29 ± 0.14
	Eudicot subfamily 2	Eudicot subfamily 3	0.26 ± 0.11
		Grass subfamily 1	0.02 ± 0.11
		Grass subfamily 2	0.17 ± 0.11
		Grass subfamily 3	0.28 ± 0.11
		Grass subfamily 4	0.26 ± 0.11
		Grass subfamily 5	0.29 ± 0.11
	Eudicot subfamily 3	Grass subfamily 1	0.32 ± 0.08
		Grass subfamily 2	0.05 ± 0.07
		Grass subfamily 3	0.17 ± 0.09
		Grass subfamily 4	0.14 ± 0.08
		Grass subfamily 5	0.14 ± 0.08
	Grass subfamily 1	Grass subfamily 2	0.35 ± 0.06
		Grass subfamily 3	0.38 ± 0.08
		Grass subfamily 4	0.34 ± 0.07
		Grass subfamily 5	0.38 ± 0.07
	Grass subfamily 2	Grass subfamily 3	0.19 ± 0.08
		Grass subfamily 4	0.18 ± 0.07
		Grass subfamily 5	0.19 ± 0.06
	Grass subfamily 3	Grass subfamily 4	0.21 ± 0.08
		Grass subfamily 5	0.22 ± 0.08
	Grass subfamily 4	Grass subfamily 5	0.08 ± 0.07
