# Supplementary material for: Divergent Evolutionary Pattern of Sugar Transporter Genes is Associated with the Difference in Sugar Accumulation between Grasses and Eudicots
Source: Sci Rep. 2016 Jun 30;6:29153. doi: 10.1038/srep29153 (PMC4928125; doi:10.1038/srep29153)

**Supporting Information for**

Divergent Evolutionary Pattern of Sugar Transporter Genes Is Associated with the Difference in sugar accumulation between Grasses and Eudicots

Wei Wang^1^, Hui Zhou^1,2^, Baiquan Ma^1,2^, Albert Owiti^1,2^ Schuyler S. Korban^3^, Yuepeng Han^1,4✝^

^1^Key Laboratory of Plant Germplasm Enhancement and Specialty Agriculture, Wuhan Botanical Garden of the Chinese Academy of Sciences, Wuhan, 430074, China

^2^Graduate University of Chinese Academy of Sciences, 19A Yuquanlu, Beijing, 100049, China

^3^Department of Biology, University of Massachusetts Boston, Boston, MA 02184, USA

^4^Sino-African Joint Research Center, Chinese Academy of Sciences, Wuhan, 430074, China

^^Corresponding author: Yuepeng Han; E-mail: yphan@wbgcas.cn; Tel/Fax: 86-27-8751-0872

Postal address: Wuhan Botanical Garden of the Chinese Academy of Sciences, Wuhan, 430074, P.R. China

E-mail addresses:

Wei Wang: ww9937@gmail.com

Hui Zhou: huichou1987@126.com

Baiquan Ma: mabaiquan10@mails.ucas.ac.cn

Albert Owiti: owitialbert@gmail.com

Schuyler S Korban: Schuyler.Korban@umb.edu

Yuepeng Han: yphan@wbgcas.cn

**Supplementary material legends**

Supplementary Table S1. Significance test for ω value between grass and eudicot subfamilies.

Supplementary Table S2. Estimation of type II functional divergence (θ) using the Type II of DIVERGE software

Supplementary Figure S1. The Clusters of Orthologous Groups (COGs) of the gene families considered in this study.

Supplementary Figure S2. Phylogenetic relationship of sequences within each gene family tested in this study. The gene tree was constructed using the neighbor joining method, and bootstrap support above 50% is shown. Red and blue points indicate recent and old gene duplication events, respectively. The dash line indicates gene loss.

Supplementary Table S1. Significance test for ω value between grass and eudicot subfamilies

| **Gene family** | **subfamily** | **mean** | ***P*-Vaule*** |
| --- | --- | --- | --- |
| HK | eudicot subfamily 1 | 0.12 ± 0.02 |  |
|  | grass subfamily 2 | 0.17 ± 0.01 | 0.1 |
|  | eudicot subfamily 2 | 0.14 ± 0.01 |  |
|  | grass subfamily 4 | 0.14 ± 0.01 | 0.9 |
|  | eudicot subfamily 3 | 0.11 ± 0.004 |  |
|  | grass subfamily 7 | 0.11 ± 0.01 | 0.7 |
|  | eudicot subfamily 4 | 0.11 ± 0.01 |  |
|  | grass subfamily 8 | 0.14 ± 0.01 | 0.002 |
|  | Grass subfamily 1 | 0.34 ± 0.01 |  |
|  | Grass subfamily 3 | 0.17 ± 0.01 |  |
|  | Grass subfamily 5 | 0.15 ± 0.01 |  |
|  | Grass subfamily 6 | 0.09 ± 0.003 |  |
|  | Grass subfamily 9 | 0.22 ± 0.004 |  |
| NI | Eudicot subfamily 1 | 0.09 ± 0.004 |  |
|  | Grass subfamily 1 | 0.08 ± 0.009 | 0.9 |
|  | Eudicot subfamily 2 | 0.11 ± 0.01 |  |
|  | Grass subfamily 2 | 0.11 ± 0.007 | 0.6 |
|  | Eudicot subfamily 3 | 0.06 ± 0.003 |  |
|  | Grass subfamily 4 | 0.07 ± 0.006 | 0.2 |
|  | Eudicot subfamily 5 | 0.05 ± 0.004 |  |
|  | Grass subfamily 8 | 0.03 ± 0.004 | 0.1 |
|  | Eudicot subfamily 4 | 0.06 ± 0.01 |  |
|  | Grass subfamily 3 | 0.15 ± 0.004 |  |
|  | Grass subfamily 5 | 0.13 ± 0.01 |  |
|  | Grass subfamily 6 | 0.03 ± 0.003 |  |
|  | Grass subfamily 7 | 0.18 ± 0.02 |  |
| SPS | Eudicot subfamily 1 | 0.15 ± 0.03 |  |
|  | Grass subfamily 1 | 0.16 ± 0.01 | 0.4 |
|  | Eudicot subfamily 2 | 0.09 ± 0.004 |  |
|  | Grass subfamily 3 | 0.09 ± 0.007 | 0.6 |
|  | Eudicot subfamily 3 | 0.08 ± 0.006 |  |
|  | Grass subfamily 4 | 0.09 ± 0.002 | 0.1 |
|  | Eudicot subfamily 4 | 0.11 ± 0.003 |  |
|  | Grass subfamily 5 | 0.12 ± 0.006 | 0.4 |
| SUSY | Eudicot subfamily 1 | 0.07 ± 0.006 |  |
|  | Grass subfamily 3 | 0.05 ± 0.003 | 0.06 |
|  | Eudicot subfamily 3 | 0.06 ± 0.002 |  |
|  | Grasse subfamily 4 | 0.06 ± 0.005 | 0.3 |
|  | Eudicot subfamily 4 | 0.07 ± 0.005 |  |
|  | Grass subfamily 6 | 0.06 ± 0.0009 | 0.1 |
|  | Eudicot subfamily 2 | 0.12 ± 0.01 |  |
|  | Grass subfamily 1 | 0.04 ± 0.01 |  |
|  | Grass subfamily 2 | 0.05 ± 0.003 |  |
|  | Grass subfamily 5 | 0.18 ± 0.02 |  |
| SUT | Eudicot subfamily 2 | 0.12 ± 0.008 |  |
|  | Grass subfamily 1 | 0.16 ± 0.01 | 0.02 |
|  | Eudicot subfamily 3 | 0.14 ± 0.007 |  |
|  | Grass subfamily 2 | 0.24 ± 0.02 | 0.00 |
|  | Eudicot subfamily 1 | 0.16 ± 0.02 |  |
|  | Grass subfamily 3 | 0.16 ± 0.008 |  |
|  | Grass subfamily 4 | 0.14 ± 0.04 |  |
|  | Grass subfamily 5 | 0.13 ± 0.009 |  |
| STP | Eudicot subfamily 2 | 0.15 ± 0.02 |  |
|  | Grass subfamily 1 | 0.09 ± 0.008 | 0.02 |
|  | Eudicot subfamily 3 | 0.08 ± 0.005 |  |
|  | Grass subfamily 6 | 0.12 ± 0.04 | 0.03 |
|  | Eudicot subfamily 6 | 0.07 ± 0.009 |  |
|  | Grass subfamily 7 | 0.05 ± 0.007 | 0.08 |
|  | Eudicot subfamily 7 | 0.12 ± 0.005 |  |
|  | Grass subfamily 8 | 0.16 ± 0.02 | 0.02 |
|  | Eudicot subfamily 8 | 0.12 ± 0.005 |  |
|  | Grass subfamily 9 | 0.27 ± 0.02 | 0.00 |
|  | Eudicot subfamily 1 | 0.06 ± 0.003 |  |
|  | Eudicot subfamily 4 | 0.07 ± 0.004 |  |
|  | Eudicot subfamily 5 | 0.09 ± 0.006 |  |
|  | Grass subfamily 2 | 0.05 ± 0.001 |  |
|  | Grass subfamily 3 | 0.07 ± 0.007 |  |
|  | Grass subfamily 4 | 0.2 ± 0.02 |  |
|  | Grass subfamily 5 | 0.13 ± 0.008 |  |
| TMT | Eudicot subfamily 1 | 0.11 ± 0.01 |  |
|  | Grass subfamily 1 | 0.09 ± 0.001 | 0.04 |
|  | Grass subfamily 2 | 0.1 ± 0.007 | 0.3 |
|  | Eudicot subfamily 3 | 0.21 ± 0.02 |  |
|  | Grass subfamily 4 | 0.13 ± 0.02 | 0.003 |
|  | Eudicot subfamily 2 | 0.09 ± 0.006 |  |
|  | Grass subfamily 3 | 0.21 ± 0.003 |  |
| VGT | Eudicot subfamily 1 | 0.19 ± 0.02 |  |
|  | Grass subfamily 1 | 0.16 ± 0.008 | 0.4 |
|  | Eudicot subfamily 2 | 0.14 ± 0.02 |  |
|  | Grass subfamily 2 | 0.22 ± 0.02 | 0.03 |
| PMT | Eudicot subfamily 1 | 0.10 ± 0.03 |  |
|  | Grass subfamily 1 | 0.12 ± 0.005 | 0.003 |
|  | Eudicot subfamily 4 | 0.09 ± 0.005 |  |
|  | Grass subfamily 4 | 0.15 ± 0.01 | 0.001 |
|  | Eudicot subfamily 2 | 0.13 ± 0.01 |  |
|  | Eudicot subfamily 3 | 0.12 ± 0.007 |  |
|  | Grass subfamily 2 | 0.25 ± 0.02 |  |
|  | Grass subfamily 3 | 0.65 ± 0.04 |  |
| INT | Eudicot subfamily 1 | 0.76 ± 0.004 |  |
|  | Grass subfamily 1 | 0.20 ± 0.02 | 0.001 |
|  | Grass subfamily 2 | 0.27 ± 0.02 | 0.001 |
|  | Eudicot subfamily 2 | 0.34 ± 0.03 |  |
|  | Grass subfamily 3 | 0.22 ± 0.01 | 0.02 |
|  | Eudicot subfamily 3 | 0.47 ± 0.03 |  |
|  | Grass subfamily 4 | 0.11 ± 0.006 | 0.001 |
| pGlcT | Eudicot subfamily 1 | 0.16 ± 0.007 |  |
|  | Grass subfamily 1 | 0.11 ± 0.02 | 0.02 |
|  | Eudicot subfamily 2 | 0.18 ± 0.009 |  |
|  | Grass subfamily 1 | 0.11 ± 0.02 | 0.007 |
|  | Eudicot subfamily 3 | 0.17 ± 0.02 |  |
|  | Grass subfamily 2 | 0.11 ± 0.02 | 0.01 |
|  | Eudicot subfamily 4 | 0.12 ± 0.01 |  |
|  | Grass subfamily 3 | 0.21 ± 0.008 | 0.000 |
| ERD6-like | Eudicot subfamily 1 | 0.61 ± 0.02 |  |
|  | Grasse subfamily 1 | 0.14 ± 0.005 | 0.000 |
|  | Eudicot subfamily 1 | 0.61 ± 0.02 |  |
|  | Grass subfamily 2 | 0.32 ± 0.01 | 0.000 |
|  | Eudicot subfamily 4 | 0.32 ± 0.02 |  |
|  | Grass subfamily 3 | 0.16 ± 0.005 | 0.001 |
|  | Grass subfamily 4 | 0.23 ± 0.04 | 0.01 |

*: Two-tailed P-value derived from t-test for ω value between grass and eudicot subfamily subfamilies.

Table S2. Estimation of type II functional divergence (θ) using the Type II of DIVERGE software

| Gene | Subfamily I | Subfamily II | θ-II value |
| --- | --- | --- | --- |
| *HK* | Eudicot subfamily 1 | Eudicot subfamily 2 | 0.01±0.14 |
|  |  | Eudicot subfamily 3 | 0.32±0.13 |
|  |  | Eudicot subfamily 4 | 0.13±0.16 |
|  |  | Grass subfamily 1 | 0.09±0.17 |
|  |  | Grass subfamily 2 | 0.04±0.11 |
|  |  | Grass subfamily 3 | 0.07±0.11 |
|  |  | Grass subfamily 4 | 0.36±0.10 |
|  |  | Grass subfamily 5 | 0.06±0.17 |
|  |  | Grass subfamily 6 | 0.01±0.19 |
|  |  | Grass subfamily 7 | 0.06±0.11 |
|  | Eudicot subfamily 2 | Eudicot subfamily 3 | 0.04±0.19 |
|  |  | Eudicot subfamily 4 | 0.11±0.15 |
|  |  | Grass subfamily 1 | 0.11±0.17 |
|  |  | Grass subfamily 2 | 0.05±0.11 |
|  |  | Grass subfamily 3 | 0.13±0.12 |
|  |  | Grass subfamily 4 | 0.32±0.10 |
|  |  | Grass subfamily 5 | 0.06±0.17 |
|  |  | Grass subfamily 6 | 0.01±0.20 |
|  |  | Grass subfamily 7 | 0.07±0.11 |
|  | Eudicot subfamily 3 | Eudicot subfamily 4 | 0.21±0.16 |
|  |  | Grass subfamily 1 | 0.02±0.20 |
|  |  | Grass subfamily 2 | 0.07±0.15 |
|  |  | Grass subfamily 3 | 0.06±0.17 |
|  |  | Grass subfamily 4 | 0.37±0.12 |
|  |  | Grass subfamily 5 | 0.05±0.18 |
|  |  | Grass subfamily 6 | 0.04±0.20 |
|  |  | Grass subfamily 7 | 0.05±0.15 |
|  | Eudicot subfamily 4 | Grass subfamily 1 | 0.18±0.17 |
|  |  | Grass subfamily 2 | 0.38±0.11 |
|  |  | Grass subfamily 3 | 0.23±0.13 |
|  |  | Grass subfamily 4 | 0.35±0.11 |
|  |  | Grass subfamily 5 | 0.17±0.17 |
|  |  | Grass subfamily 6 | 0.19±0.18 |
|  |  | Grass subfamily 7 | 0.17±0.10 |
|  | Grass subfamily 1 | Grass subfamily 2 | 0.08±0.16 |
|  |  | Grass subfamily 3 | 0.11±0.16 |
|  |  | Grass subfamily 4 | 0.31±0.12 |
|  |  | Grass subfamily 5 | 0.11±0.21 |
|  |  | Grass subfamily 6 | 0.08±0.23 |
|  |  | Grass subfamily 7 | 0.04±0.16 |
|  | Grass subfamily 2 | Grass subfamily 3 | 0.02±0.06 |
|  |  | Grass subfamily 4 | 0.35±0.08 |
|  |  | Grass subfamily 5 | 0.10±0.15 |
|  |  | Grass subfamily 6 | 0.04±0.18 |
|  |  | Grass subfamily 7 | 0.04±0.07 |
|  | Grass subfamily 3 | Grass subfamily 4 | 0.29±0.09 |
|  |  | Grass subfamily 5 | 0.10±0.15 |
|  |  | Grass subfamily 6 | 0.09±0.18 |
|  |  | Grass subfamily 7 | 0.04±0.07 |
|  | Grass subfamily 4 | Grass subfamily 5 | 0.30±0.11 |
|  |  | Grass subfamily 6 | 0.21±0.13 |
|  |  | Grass subfamily 7 | 0.37±0.09 |
|  | Grass subfamily 5 | Grass subfamily 6 | 0.05±0.27 |
|  |  | Grass subfamily 7 | 0.01±0.15 |
|  | Grass subfamily 6 | Grass subfamily 7 | 0.04±0.18 |
| *NI* | Eudicot subfamily 1 | Eudicot subfamily 2 | 0.02±0.04 |
|  |  | Eudicot subfamily 3 | 0.09±0.05 |
|  |  | Eudicot subfamily 4 | 0.27±0.05 |
|  |  | Eudicot subfamily 5 | 0.27±0.05 |
|  |  | Grass subfamily 1 | 0.01±0.37 |
|  |  | Grass subfamily 2 | 0.09±0.65 |
|  |  | Grass subfamily 3 | 0.01±0.05 |
|  |  | Grass subfamily 4 | 0.25±0.05 |
|  |  | Grass subfamily 5 | 0.28±0.04 |
|  |  | Grass subfamily 6 | 0.29±0.04 |
|  |  | Grass subfamily 7 | 0.19±0.05 |
|  | Eudicot subfamily 2 | Eudicot subfamily 3 | 0.05±0.05 |
|  |  | Eudicot subfamily 4 | 0.29±0.05 |
|  |  | Eudicot subfamily 5 | 0.26±0.05 |
|  |  | Grass subfamily 1 | 0.02±0.13 |
|  |  | Grass subfamily 2 | 0.01±0.18 |
|  |  | Grass subfamily 3 | 0.04±0.04 |
|  |  | Grass subfamily 4 | 0.30±0.05 |
|  |  | Grass subfamily 5 | 0.30±0.04 |
|  |  | Grass subfamily 6 | 0.29±0.04 |
|  |  | Grass subfamily 7 | 0.26±0.05 |
|  | Eudicot subfamily 3 | Eudicot subfamily 4 | 0.28±0.05 |
|  |  | Eudicot subfamily 5 | 0.27±0.05 |
|  |  | Grass subfamily 1 | 0.04±0.07 |
|  |  | Grass subfamily 2 | 0.02±0.04 |
|  |  | Grass subfamily 3 | 0.01±0.04 |
|  |  | Grass subfamily 4 | 0.33±0.04 |
|  |  | Grass subfamily 5 | 0.33±0.04 |
|  |  | Grass subfamily 6 | 0.33±0.04 |
|  |  | Grass subfamily 7 | 0.24±0.05 |
|  | Eudicot subfamily 4 | Eudicot subfamily 5 | 0.02±0.05 |
|  |  | Grass subfamily 1 | 0.05±0.07 |
|  |  | Grass subfamily 2 | 0.02±0.08 |
|  |  | Grass subfamily 3 | 0.25±0.05 |
|  |  | Grass subfamily 4 | 0.13±0.05 |
|  |  | Grass subfamily 5 | 0.06±0.04 |
|  |  | Grass subfamily 6 | 0.05±0.04 |
|  |  | Grass subfamily 7 | 0.04±0.05 |
|  | Eudicot subfamily 5 | Grass subfamily 1 | 0.04±0.07 |
|  |  | Grass subfamily 2 | 0.02±0.07 |
|  |  | Grass subfamily 3 | 0.27±0.04 |
|  |  | Grass subfamily 4 | 0.13±0.04 |
|  |  | Grass subfamily 5 | 0.08±0.04 |
|  |  | Grass subfamily 6 | 0.06±0.04 |
|  |  | Grass subfamily 7 | 0.05±0.05 |
|  | Grass subfamily 1 | Grass subfamily 2 | 0.09±0.19 |
|  |  | Grass subfamily 3 | 0.02±0.04 |
|  |  | Grass subfamily 4 | 0.02±0.05 |
|  |  | Grass subfamily 5 | 0.01±0.08 |
|  |  | Grass subfamily 6 | 0.01±0.04 |
|  |  | Grass subfamily 7 | 0.01±0.05 |
|  | Grass subfamily 2 | Grass subfamily 3 | 0.01±0.02 |
|  |  | Grass subfamily 4 | 0.01±0.03 |
|  |  | Grass subfamily 5 | 0.01±0.02 |
|  |  | Grass subfamily 6 | 0.01±0.03 |
|  |  | Grass subfamily 7 | 0.01±0.03 |
|  | Grass subfamily 3 | Grass subfamily 4 | 0.33±0.04 |
|  |  | Grass subfamily 5 | 0.37±0.04 |
|  |  | Grass subfamily 6 | 0.36±0.04 |
|  |  | Grass subfamily 7 | 0.24±0.05 |
|  | Grass subfamily 4 | Grass subfamily 5 | 0.14±0.04 |
|  |  | Grass subfamily 6 | 0.15±0.04 |
|  |  | Grass subfamily 7 | 0.08±0.05 |
|  | Grass subfamily 5 | Grass subfamily 6 | 0.10±0.03 |
|  |  | Grass subfamily 7 | 0.06±0.04 |
|  | Grass subfamily 6 | Grass subfamily 7 | 0.07±0.04 |
| *SUSY* | Eudicot subfamily 1 | Eudicot subfamily 2 | 0.10±0.05 |
|  |  | Eudicot subfamily 3 | 0.16±0.05 |
|  |  | Eudicot subfamily 4 | 0.07±0.07 |
|  |  | Grass subfamily 1 | 0.01±0.05 |
|  |  | Grass subfamily 2 | 0.01±0.05 |
|  |  | Grass subfamily 3 | 0.12±0.05 |
|  |  | Grass subfamily 4 | 0.16±0.06 |
|  |  | Grass subfamily 5 | 0.17±0.05 |
|  | Eudicot subfamily 2 | Eudicot subfamily 3 | 0.01±0.04 |
|  |  | Eudicot subfamily 4 | 0.05±0.07 |
|  |  | Grass subfamily 1 | 0.06±0.04 |
|  |  | Grass subfamily 2 | 0.09±0.04 |
|  |  | Grass subfamily 3 | 0.01±0.04 |
|  |  | Grass subfamily 4 | 0.14±0.05 |
|  |  | Grass subfamily 5 | 0.18±0.04 |
|  | Eudicot subfamily 3 | Eudicot subfamily 4 | 0.10±0.07 |
|  |  | Grass subfamily 1 | 0.13±0.04 |
|  |  | Grass subfamily 2 | 0.18±0.04 |
|  |  | Grass subfamily 3 | 0.05±0.04 |
|  |  | Grass subfamily 4 | 0.22±0.05 |
|  |  | Grass subfamily 5 | 0.24±0.04 |
|  | Eudicot subfamily 4 | Grass subfamily 1 | 0.08±0.07 |
|  |  | Grass subfamily 2 | 0.06±0.07 |
|  |  | Grass subfamily 3 | 0.07±0.07 |
|  |  | Grass subfamily 4 | 0.05±0.07 |
|  |  | Grass subfamily 5 | 0.04±0.07 |
|  | Grass subfamily 1 | Grass subfamily 2 | 0.08±0.03 |
|  |  | Grass subfamily 3 | 0.18±0.03 |
|  |  | Grass subfamily 4 | 0.16±0.05 |
|  |  | Grass subfamily 5 | 0.28±0.03 |
|  | Grass subfamily 2 | Grass subfamily 3 | 0.20±0.03 |
|  |  | Grass subfamily 4 | 0.14±0.05 |
|  |  | Grass subfamily 5 | 0.30±0.03 |
|  | Grass subfamily 3 | Grass subfamily 4 | 0.12±0.05 |
|  |  | Grass subfamily 5 | 0.25±0.03 |
|  | Grass subfamily 4 | Grass subfamily 5 | 0.04±0.05 |
| *SPS* | Eudicot subfamily 1 | Eudicot subfamily 2 | 0.30±0.02 |
|  |  | Eudicot subfamily 3 | 0.30±0.04 |
|  |  | Eudicot subfamily 4 | 0.29±0.06 |
|  |  | Grass subfamily 1 | 0.06±0.07 |
|  |  | Grass subfamily 2 | 0.24±0.05 |
|  |  | Grass subfamily 3 | 0.14±0.04 |
|  |  | Grass subfamily 4 | 0.15±0.02 |
|  |  | Grass subfamily 5 | 0.10±0.03 |
|  | Eudicot subfamily 2 | Eudicot subfamily 3 | 0.19±0.05 |
|  |  | Eudicot subfamily 4 | 0.27±0.05 |
|  |  | Grass subfamily 1 | 0.07±0.05 |
|  |  | Grass subfamily 2 | 0.03±0.09 |
|  |  | Grass subfamily 3 | 0.38±0.09 |
|  |  | Grass subfamily 4 | 0.23±0.11 |
|  |  | Grass subfamily 5 | 0.35±0.07 |
|  | Eudicot subfamily 3 | Eudicot subfamily 4 | 0.17±0.11 |
|  |  | Grass subfamily 1 | 0.16±0.02 |
|  |  | Grass subfamily 2 | 0.22±0.07 |
|  |  | Grass subfamily 3 | 0.18±0.05 |
|  |  | Grass subfamily 4 | 0.04±0.05 |
|  |  | Grass subfamily 5 | 0.25±0.05 |
|  | Eudicot subfamily 4 | Grass subfamily 1 | 0.13±0.05 |
|  |  | Grass subfamily 2 | 0.06±0.02 |
|  |  | Grass subfamily 3 | 0.05±0.02 |
|  |  | Grass subfamily 4 | 0.11±0.03 |
|  |  | Grass subfamily 5 | 0.01±0.04 |
|  | Grass subfamily 1 | Grass subfamily 2 | 0.04±0.07 |
|  |  | Grass subfamily 3 | 0.19±0.04 |
|  |  | Grass subfamily 4 | 0.05±0.05 |
|  |  | Grass subfamily 5 | 0.29±0.03 |
|  | Grass subfamily 2 | Grass subfamily 3 | 0.26±0.07 |
|  |  | Grass subfamily 4 | 0.02±0.13 |
|  |  | Grass subfamily 5 | 0.01±0.18 |
|  | Grass subfamily 3 | Grass subfamily 4 | 0.07±0.04 |
|  |  | Grass subfamily 5 | 0.30±0.05 |
|  | Grass subfamily 4 | Grass subfamily 5 | 0.30±0.04 |
| *ERD6-like* | Eudicot subfamily 1 | Eudicot subfamily 2 | 0.31±0.07 |
|  |  | Eudicot subfamily 3 | 0.21±0.07 |
|  |  | Grass subfamily 1 | 0.14±0.07 |
|  |  | Grass subfamily 2 | 0.20±0.08 |
|  |  | Grass subfamily 3 | 0.21±0.09 |
|  |  | Grass subfamily 4 | 0.23±0.09 |
|  | Eudicot subfamily 2 | Eudicot subfamily 3 | 0.21±0.05 |
|  |  | Grass subfamily 1 | 0.04±0.06 |
|  |  | Grass subfamily 2 | 0.02±0.04 |
|  |  | Grass subfamily 3 | 0.32±0.07 |
|  |  | Grass subfamily 4 | 0.37±0.05 |
|  | Eudicot subfamily 3 | Grass subfamily 1 | 0.02±0.09 |
|  |  | Grass subfamily 2 | 0.30±0.04 |
|  |  | Grass subfamily 3 | 0.04±0.10 |
|  |  | Grass subfamily 4 | 0.03±0.07 |
|  | Grass subfamily 1 | Grass subfamily 2 | 0.05±0.07 |
|  |  | Grass subfamily 3 | 0.46±0.05 |
|  |  | Grass subfamily 4 | 0.40±0.06 |
|  | Grass subfamily 2 | Grass subfamily 3 | 0.34±0.07 |
|  |  | Grass subfamily 4 | 0.41±0.07 |
|  | Grass subfamily 3 | Grass subfamily 4 | 0.02±0.11 |
| *INT* | Eudicot subfamily1 | Eudicot subfamily2 | 0.27±0.11 |
|  |  | Eudicot subfamily3 | 0.18±0.06 |
|  |  | Grass subfamily 1 | 0.04±0.10 |
|  |  | Grass subfamily 2 | 0.20±0.12 |
|  |  | Grass subfamily 3 | 0.22±0.12 |
|  | Eudicot subfamily2 | Eudicot subfamily3 | 0.33±0.09 |
|  |  | Grass subfamily 1 | 0.32±0.05 |
|  |  | Grass subfamily 2 | 0.02±0.07 |
|  |  | Grass subfamily 3 | 0.34±0.07 |
|  | Eudicot subfamily3 | Grass subfamily 1 | 0.41±0.07 |
|  |  | Grass subfamily 2 | 0.37±0.05 |
|  |  | Grass subfamily 3 | 0.02±0.09 |
|  | Grass subfamily 1 | Grass subfamily 2 | 0.30±0.04 |
|  |  | Grass subfamily 3 | 0.40±0.06 |
|  | Grass subfamily 2 | Grass subfamily 3 | 0.46±0.05 |
| *pGlcT* | Eudicot subfamily 1 | Eudicot subfamily 2 | 0.31±0.11 |
|  |  | Eudicot subfamily 3 | 0.31±0.12 |
|  |  | Grass subfamily 1 | 0.04±0.12 |
|  |  | Grass subfamily 2 | 0.24±0.12 |
|  |  | Grass subfamily 3 | 0.26±0.12 |
|  | Eudicot subfamily 2 | Eudicot subfamily 3 | 0.39±0.09 |
|  |  | Grass subfamily 1 | 0.42±0.07 |
|  |  | Grass subfamily 2 | 0.02±0.07 |
|  |  | Grass subfamily 3 | 0.44±0.07 |
|  | Eudicot subfamily 3 | Grass subfamily 1 | 0.39±0.08 |
|  |  | Grass subfamily 2 | 0.35±0.09 |
|  |  | Grass subfamily 3 | 0.02±0.09 |
|  | Grass subfamily 1 | Grass subfamily 2 | 0.37±0.07 |
|  |  | Grass subfamily 3 | 0.45±0.06 |
|  | Grass subfamily 2 | Grass subfamily 3 | 0.43±0.07 |
| *PMT* | Eudicot subfamily 1 | Eudicot subfamily 2 | 0.18±0.13 |
|  |  | Eudicot subfamily 3 | 0.21±0.12 |
|  |  | Eudicot subfamily 4 | 0.11±0.11 |
|  |  | Grass subfamily 1 | 0.11±0.12 |
|  |  | Grass subfamily 2 | 0.12±0.12 |
|  |  | Grass subfamily 3 | 0.22±0.11 |
|  | Eudicot subfamily 2 | Eudicot subfamily 3 | 0.23±0.10 |
|  |  | Eudicot subfamily 4 | 0.17±0.09 |
|  |  | Grass subfamily 1 | 0.16±0.09 |
|  |  | Grass subfamily 2 | 024±0.09 |
|  |  | Grass subfamily 3 | 0.19±0.09 |
|  | Eudicot subfamily 3 | Eudicot subfamily 4 | 0.21±0.08 |
|  |  | Grass subfamily 1 | 0.19±0.08 |
|  |  | Grass subfamily 2 | 0.24±0.08 |
|  |  | Grass subfamily 3 | 0.02±0.08 |
|  | Eudicot subfamily 4 | Grass subfamily 1 | 0.16±0.06 |
|  |  | Grass subfamily 2 | 0.24±0.06 |
|  |  | Grass subfamily 3 | 0.34±0.06 |
|  | Grass subfamily 1 | Grass subfamily 2 | 0.10±0.06 |
|  |  | Grass subfamily 3 | 0.32±0.06 |
|  | Grass subfamily 2 | Grass subfamily 3 | 0.41±0.06 |
| *VGT* | Eudicot subfamily 1 | Eudicot subfamily 2 | 0.09±0.07 |
|  |  | Grass subfamily 1 | 0.05±0.06 |
|  |  | Grass subfamily 2 | 0.13±0.07 |
|  | Eudicot subfamily 2 | Grass subfamily 1 | 0.14±0.06 |
|  |  | Grass subfamily 2 | 0.01±0.06 |
|  | Grass subfamily 1 | Grass subfamily 2 | 0.20±0.05 |
| *TMT* | Eudicot subfamily 1 | Eudicot subfamily 2 | 0.02±0.08 |
|  |  | Eudicot subfamily 3 | 0.25±0.05 |
|  |  | Grass subfamily 1 | 0.03±0.05 |
|  |  | Grass subfamily 2 | 0.04±0.04 |
|  |  | Grass subfamily 3 | 0.15±0.04 |
|  |  | Grass subfamily 4 | 0.24±0.02 |
|  |  | Grass subfamily 5 | 0.21±0.07 |
|  | Eudicot subfamily 2 | Eudicot subfamily 3 | 0.22±0.07 |
|  |  | Grass subfamily 1 | 0.07±0.07 |
|  |  | Grass subfamily 2 | 0.03±0.04 |
|  |  | Grass subfamily 3 | 0.18±0.01 |
|  |  | Grass subfamily 4 | 0.26±0.04 |
|  |  | Grass subfamily 5 | 0.15±0.04 |
|  | Eudicot subfamily 3 | Grass subfamily 1 | 0.19±0.09 |
|  |  | Grass subfamily 2 | 0.12±0.04 |
|  |  | Grass subfamily 3 | 0.20±0.05 |
|  |  | Grass subfamily 4 | 0.10±0.02 |
|  |  | Grass subfamily 5 | 0.11±0.04 |
|  | Grass subfamily 1 | Grass subfamily 2 | 0.01±0.05 |
|  |  | Grass subfamily 3 | 0.31±0.02 |
|  |  | Grass subfamily 4 | 0.20±0.07 |
|  |  | Grass subfamily 5 | 0.01±0.02 |
|  | Grass subfamily 2 | Grass subfamily 3 | 0.21±0.03 |
|  |  | Grass subfamily 4 | 0.11±0.01 |
|  |  | Grass subfamily 5 | 0.33±0.04 |
|  | Grass subfamily 3 | Grass subfamily 4 | 0.07±0.10 |
|  |  | Grass subfamily 5 | 0.36±0.04 |
|  | Grass subfamily 4 | Grass subfamily 5 | 0.24±0.05 |
| *STP* | Eudicot subfamily 1 | Eudicot subfamily 2 | 0.04±0.11 |
|  |  | Eudicot subfamily 3 | 0.25±0.05 |
|  |  | Eudicot subfamily 4 | 0.13±0.05 |
|  |  | Eudicot subfamily 5 | 0.16±0.07 |
|  |  | Eudicot subfamily 6 | 0.24±0.03 |
|  |  | Eudicot subfamily 7 | 0.17±0.04 |
|  |  | Eudicot subfamily 8 | 0.16±0.02 |
|  |  | Grass subfamily 1 | 0.02±0.05 |
|  |  | Grass subfamily 2 | 0.05±0.07 |
|  |  | Grass subfamily 3 | 0.02±0.08 |
|  |  | Grass subfamily 4 | 0.06±0.04 |
|  |  | Grass subfamily 5 | 0.05±0.04 |
|  |  | Grass subfamily 6 | 0.30±0.02 |
|  |  | Grass subfamily 7 | 0.30±0.04 |
|  |  | Grass subfamily 8 | 0.29±0.06 |
|  |  | Grass subfamily 9 | 0.06±0.07 |
|  | Eudicot subfamily 2 | Eudicot subfamily 3 | 0.24±0.03 |
|  |  | Eudicot subfamily 4 | 0.14±0.04 |
|  |  | Eudicot subfamily 5 | 0.15±0.02 |
|  |  | Eudicot subfamily 6 | 0.10±0.03 |
|  |  | Eudicot subfamily 7 | 0.19±0.05 |
|  |  | Eudicot subfamily 8 | 0.27±0.05 |
|  |  | Grass subfamily 1 | 0.07±0.05 |
|  |  | Grass subfamily 2 | 0.03±0.09 |
|  |  | Grass subfamily 3 | 0.08±0.09 |
|  |  | Grass subfamily 4 | 0.03±0.11 |
|  |  | Grass subfamily 5 | 0.05±0.07 |
|  |  | Grass subfamily 6 | 0.17±0.11 |
|  |  | Grass subfamily 7 | 0.16±0.02 |
|  |  | Grass subfamily 8 | 0.22±0.07 |
|  |  | Grass subfamily 9 | 0.18±0.05 |
|  | Eudicot subfamily 3 | Eudicot subfamily 4 | 0.14±0.05 |
|  |  | Eudicot subfamily 5 | 0.25±0.05 |
|  |  | Eudicot subfamily 6 | 0.13±0.05 |
|  |  | Eudicot subfamily 7 | 0.36±0.02 |
|  |  | Eudicot subfamily 8 | 0.15±0.02 |
|  |  | Grass subfamily 1 | 0.11±0.03 |
|  |  | Grass subfamily 2 | 0.21±0.04 |
|  |  | Grass subfamily 3 | 0.04±0.07 |
|  |  | Grass subfamily 4 | 0.19±0.04 |
|  |  | Grass subfamily 5 | 0.05±0.05 |
|  |  | Grass subfamily 6 | 0.29±0.03 |
|  |  | Grass subfamily 7 | 0.26±0.07 |
|  |  | Grass subfamily 8 | 0.02±0.13 |
|  |  | Grass subfamily 9 | 0.01±0.18 |
|  | Eudicot subfamily 4 | Eudicot subfamily 5 | 0.07±0.04 |
|  |  | Eudicot subfamily 6 | 0.30±0.05 |
|  |  | Eudicot subfamily 7 | 0.30±0.04 |
|  |  | Eudicot subfamily 8 | 0.17±0.03 |
|  |  | Grass subfamily 1 | 0.21±0.07 |
|  |  | Grass subfamily 2 | 0.14±0.02 |
|  |  | Grass subfamily 3 | 0.20±0.07 |
|  |  | Grass subfamily 4 | 0.05±0.09 |
|  |  | Grass subfamily 5 | 0.23±0.05 |
|  |  | Grass subfamily 6 | 0.21±0.01 |
|  |  | Grass subfamily 7 | 0.14±0.06 |
|  |  | Grass subfamily 8 | 0.02±0.04 |
|  |  | Grass subfamily 9 | 0.32±0.07 |
|  | Eudicot subfamily 5 | Eudicot subfamily 6 | 0.37±0.05 |
|  |  | Eudicot subfamily 7 | 0.22±0.09 |
|  |  | Eudicot subfamily 8 | 0.30±0.04 |
|  |  | Grass subfamily 1 | 0.04±0.10 |
|  |  | Grass subfamily 2 | 0.03±0.07 |
|  |  | Grass subfamily 3 | 0.05±0.07 |
|  |  | Grass subfamily 4 | 0.46±0.05 |
|  |  | Grass subfamily 5 | 0.30±0.02 |
|  |  | Grass subfamily 6 | 0.27±0.01 |
|  |  | Grass subfamily 7 | 0.29±0.06 |
|  |  | Grass subfamily 8 | 0.06±0.07 |
|  |  | Grass subfamily 9 | 0.14±0.05 |
|  | Eudicot subfamily 6 | Eudicot subfamily 7 | 0.14±0.04 |
|  |  | Eudicot subfamily 8 | 0.15±0.03 |
|  |  | Grass subfamily 1 | 0.13±0.02 |
|  |  | Grass subfamily 2 | 0.19±0.01 |
|  |  | Grass subfamily 3 | 0.27±0.05 |
|  |  | Grass subfamily 4 | 0.09±0.04 |
|  |  | Grass subfamily 5 | 0.03±0.09 |
|  |  | Grass subfamily 6 | 0.38±0.09 |
|  |  | Grass subfamily 7 | 0.23±0.08 |
|  |  | Grass subfamily 8 | 0.25±0.07 |
|  |  | Grass subfamily 9 | 0.17±0.11 |
|  | Eudicot subfamily 7 | Eudicot subfamily 8 | 0.26±0.02 |
|  |  | Grass subfamily 1 | 0.22±0.01 |
|  |  | Grass subfamily 2 | 0.18±0.05 |
|  |  | Grass subfamily 3 | 0.04±0.05 |
|  |  | Grass subfamily 4 | 0.25±0.05 |
|  |  | Grass subfamily 5 | 0.13±0.05 |
|  |  | Grass subfamily 6 | 0.26±0.02 |
|  |  | Grass subfamily 7 | 0.05±0.02 |
|  |  | Grass subfamily 8 | 0.11±0.03 |
|  |  | Grass subfamily 9 | 0.31±0.05 |
|  | Eudicot subfamily 8 | Grass subfamily 1 | 0.34±0.07 |
|  |  | Grass subfamily 2 | 0.19±0.04 |
|  |  | Grass subfamily 3 | 0.35±0.05 |
|  |  | Grass subfamily 4 | 0.29±0.03 |
|  |  | Grass subfamily 5 | 0.15±0.07 |
|  |  | Grass subfamily 6 | 0.26±0.02 |
|  |  | Grass subfamily 7 | 0.19±0.04 |
|  |  | Grass subfamily 8 | 0.21±0.01 |
|  |  | Grass subfamily 9 | 0.04±0.05 |
|  | Grass subfamily 1 | Grass subfamily 2 | 0.08±0.14 |
|  |  | Grass subfamily 3 | 0.05±0.07 |
|  |  | Grass subfamily 4 | 0.03±0.04 |
|  |  | Grass subfamily 5 | 0.04±0.08 |
|  |  | Grass subfamily 6 | 0.05±0.04 |
|  |  | Grass subfamily 7 | 0.22±0.05 |
|  |  | Grass subfamily 8 | 0.24±0.04 |
|  |  | Grass subfamily 9 | 0.18±0.07 |
|  | Grass subfamily 2 | Grass subfamily 3 | 0.06±0.07 |
|  |  | Grass subfamily 4 | 0.07±0.07 |
|  |  | Grass subfamily 5 | 0.05±0.07 |
|  |  | Grass subfamily 6 | 0.14±0.07 |
|  |  | Grass subfamily 7 | 0.38±0.03 |
|  |  | Grass subfamily 8 | 0.18±0.03 |
|  |  | Grass subfamily 9 | 0.16±0.05 |
|  | Grass subfamily 3 | Grass subfamily 4 | 0.08±0.03 |
|  |  | Grass subfamily 5 | 0.02±0.03 |
|  |  | Grass subfamily 6 | 0.14±0.05 |
|  |  | Grass subfamily 7 | 0.30±0.03 |
|  |  | Grass subfamily 8 | 0.12±0.05 |
|  |  | Grass subfamily 9 | 0.02±0.04 |
|  | Grass subfamily 4 | Grass subfamily 5 | 0.09±0.09 |
|  |  | Grass subfamily 6 | 0.27±0.05 |
|  |  | Grass subfamily 7 | 0.27±0.05 |
|  |  | Grass subfamily 8 | 0.31±0.17 |
|  |  | Grass subfamily 9 | 0.09±0.05 |
|  | Grass subfamily 5 | Grass subfamily 6 | 0.21±0.05 |
|  |  | Grass subfamily 7 | 0.25±0.05 |
|  |  | Grass subfamily 8 | 0.28±0.04 |
|  |  | Grass subfamily 9 | 0.29±0.04 |
|  | Grass subfamily 6 | Grass subfamily 7 | 0.19±0.05 |
|  |  | Grass subfamily 8 | 0.25±0.05 |
|  |  | Grass subfamily 9 | 0.19±0.05 |
|  | Grass subfamily 7 | Grass subfamily 8 | 0.26±0.02 |
|  |  | Grass subfamily 9 | 0.32±0.13 |
|  | Grass subfamily 8 | Grass subfamily 9 | 0.21±0.08 |

Supplementary Figure S1. The Clusters of Orthologous Groups (COGs) of the gene families considered in this study.

*HK*


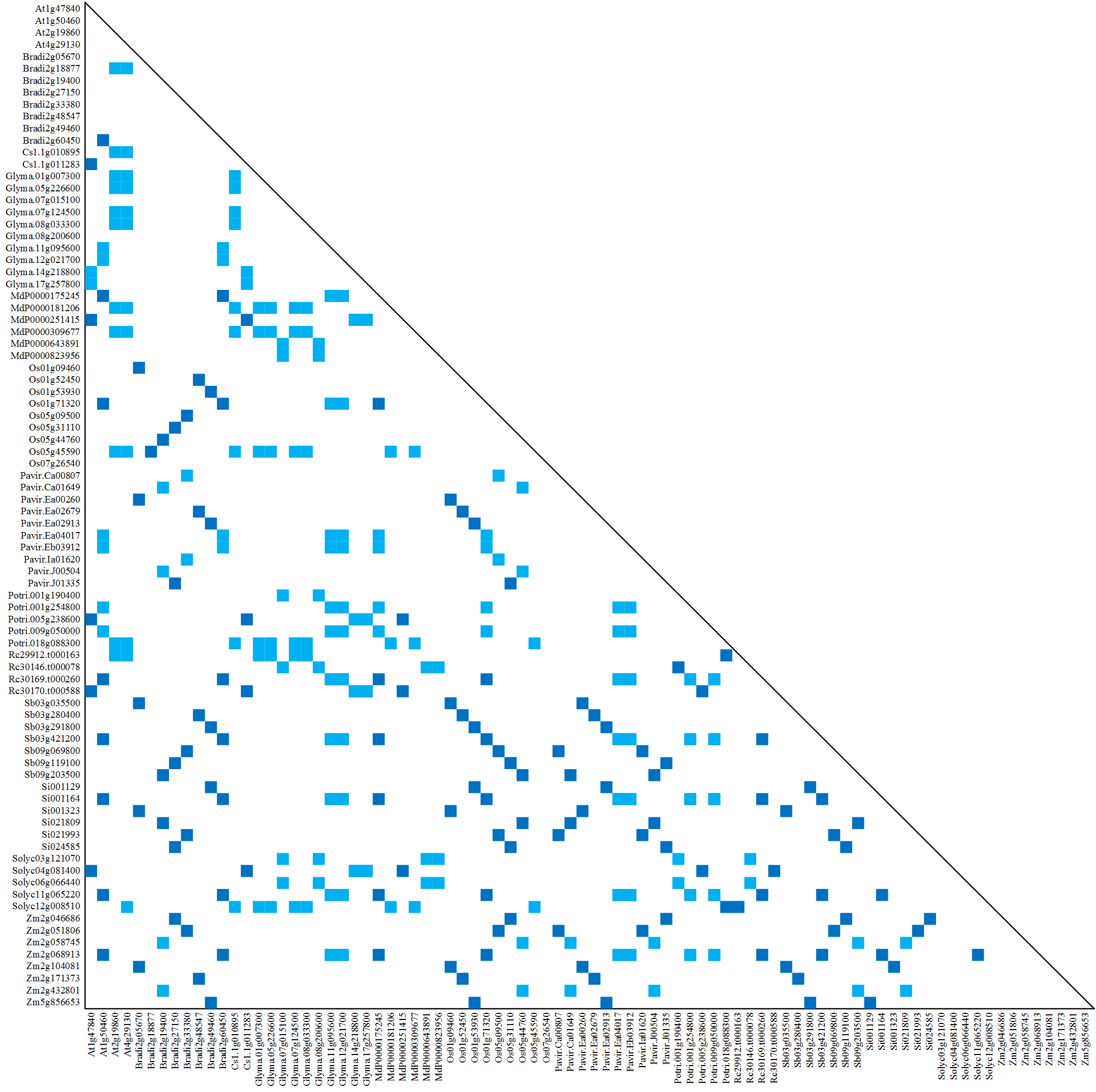


*NI*


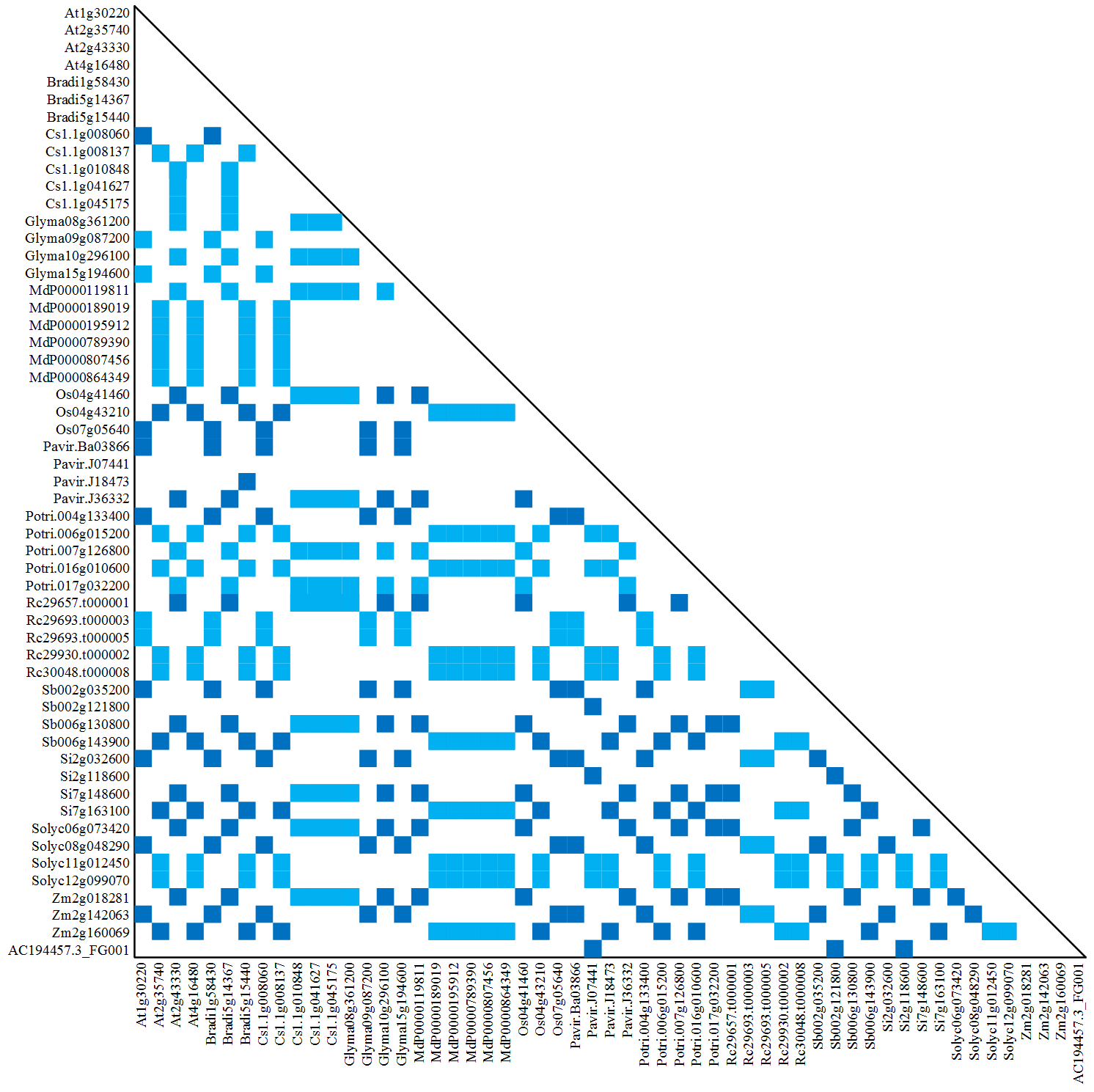


*PMT*


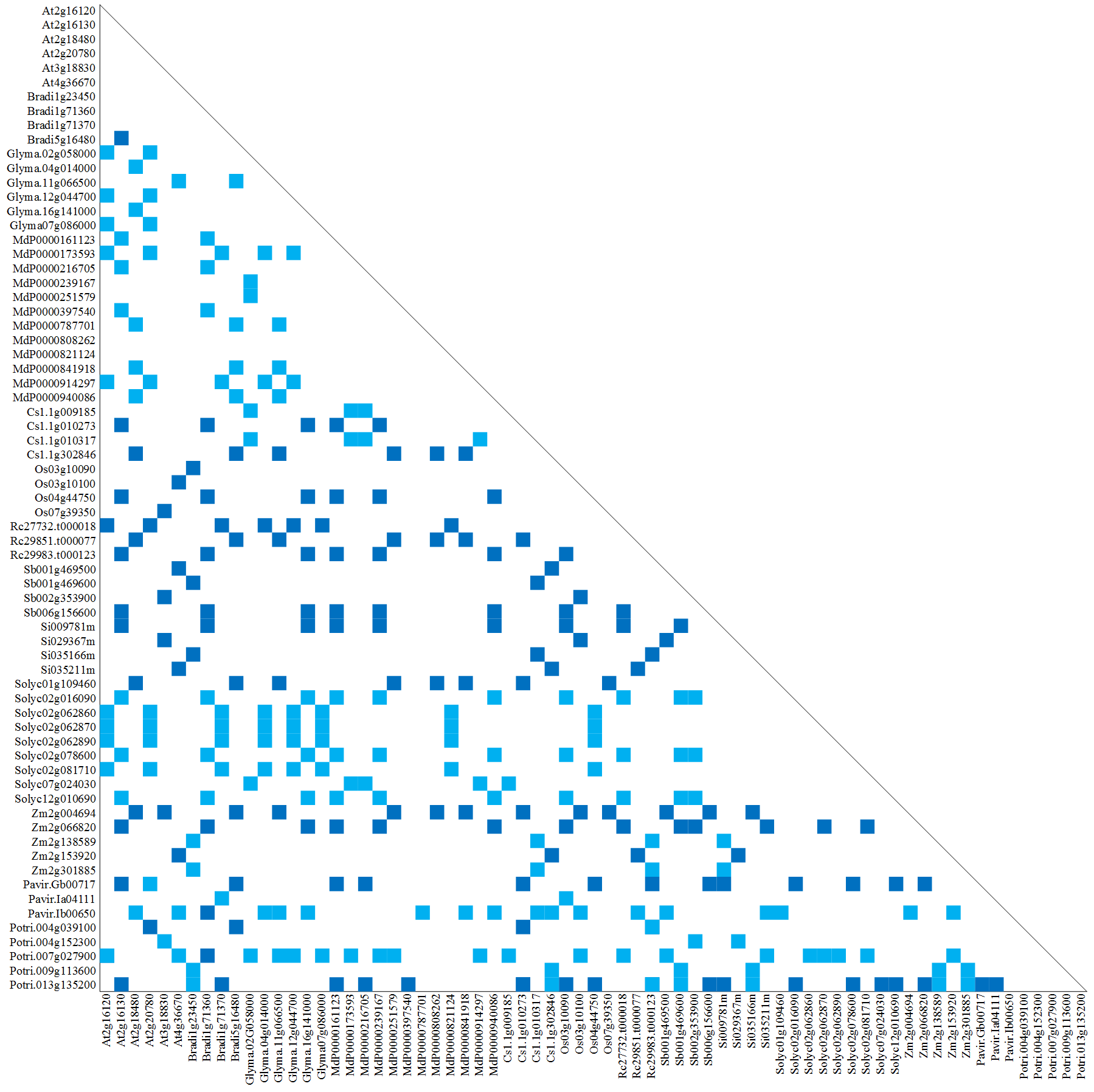


*SPS*


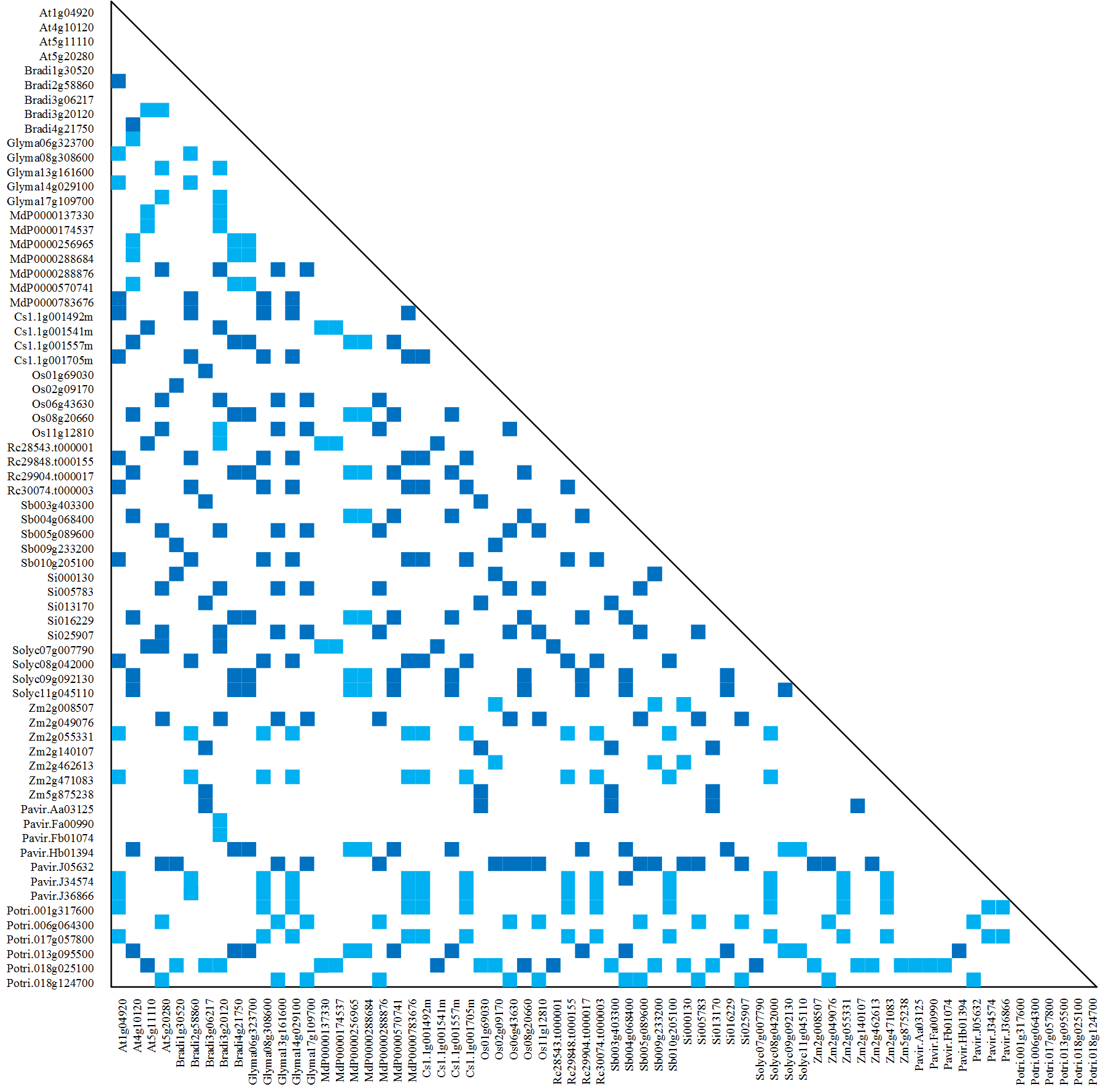


*STP*


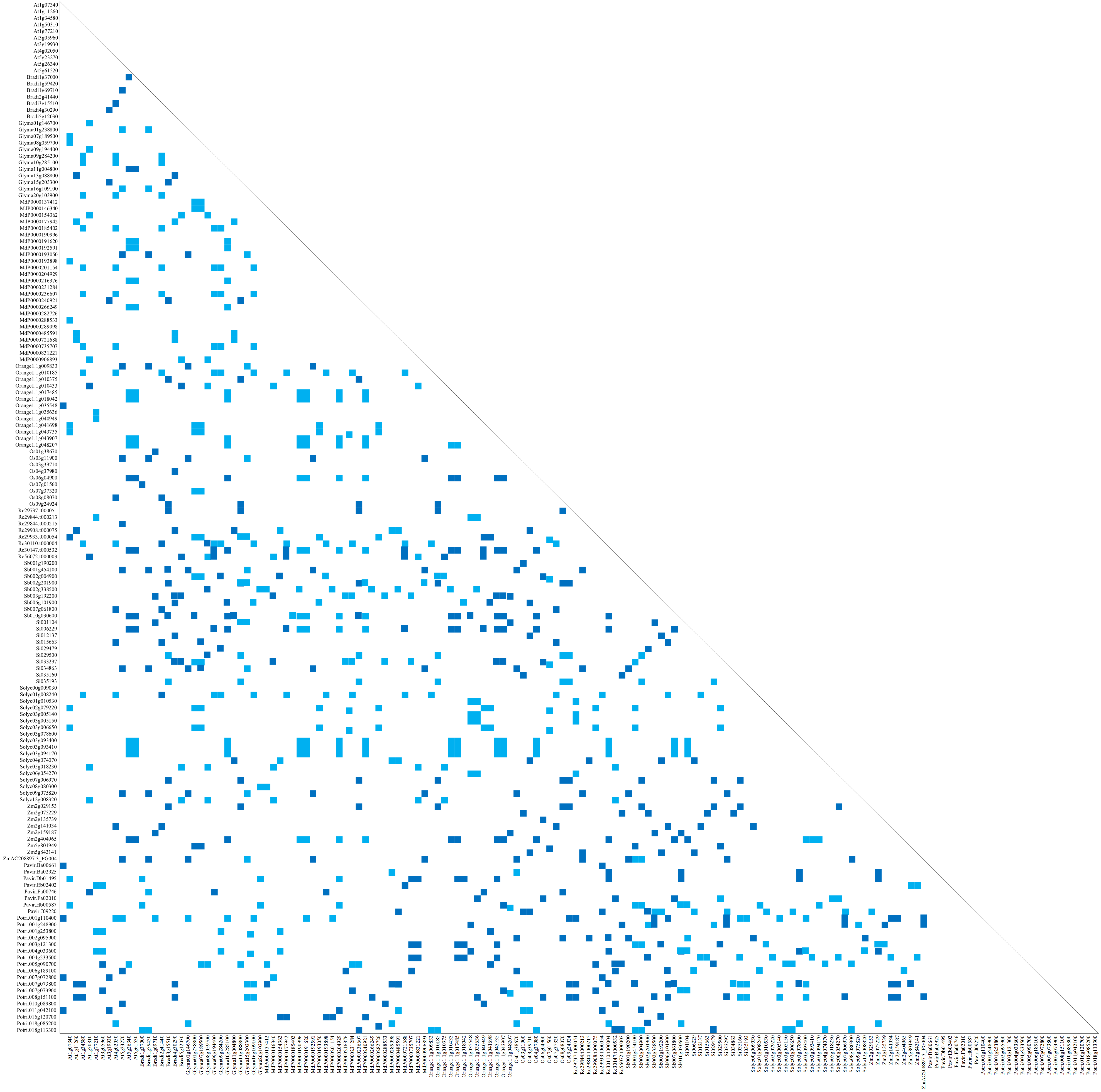


*SUSY*


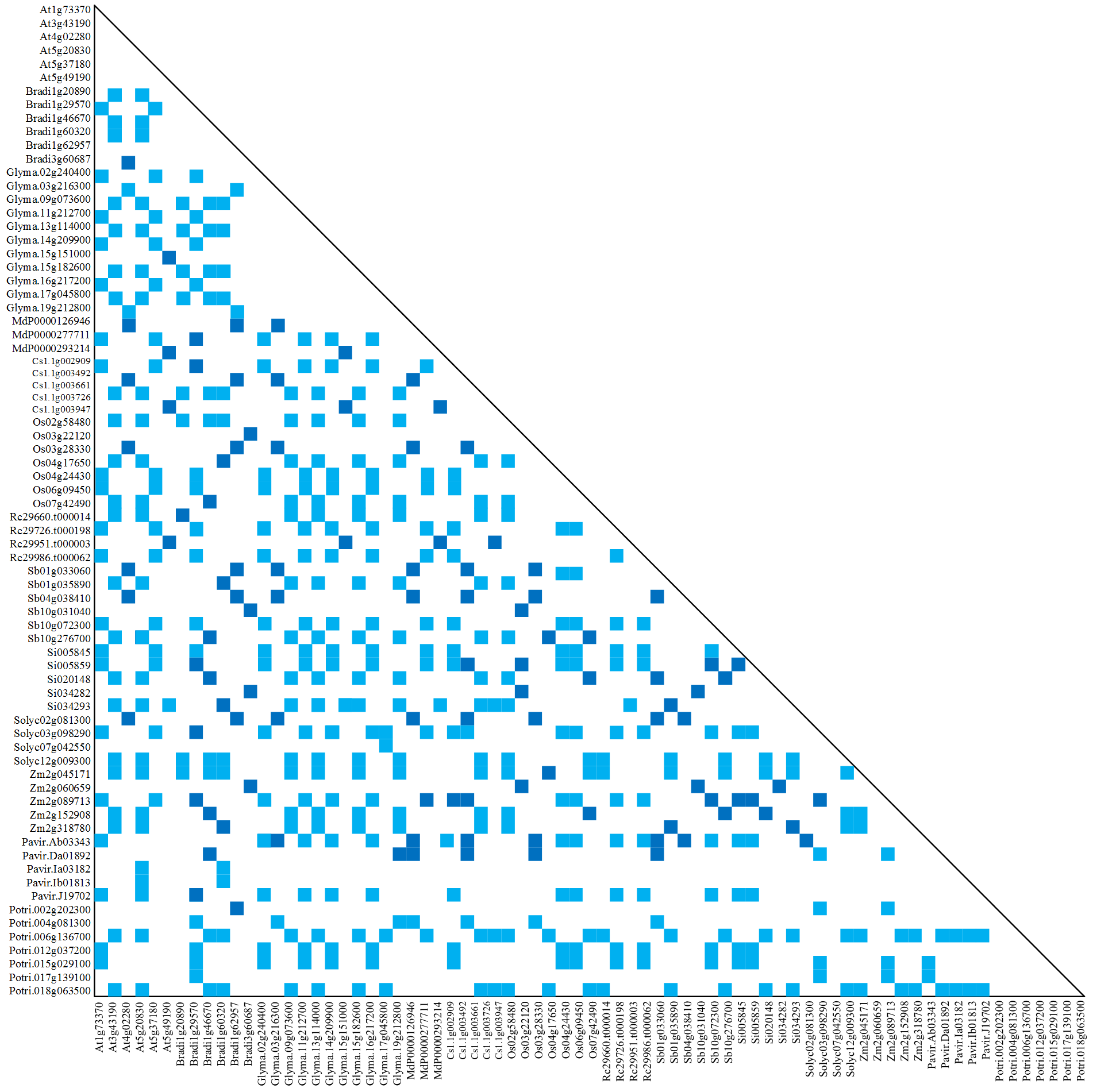


*TMT*


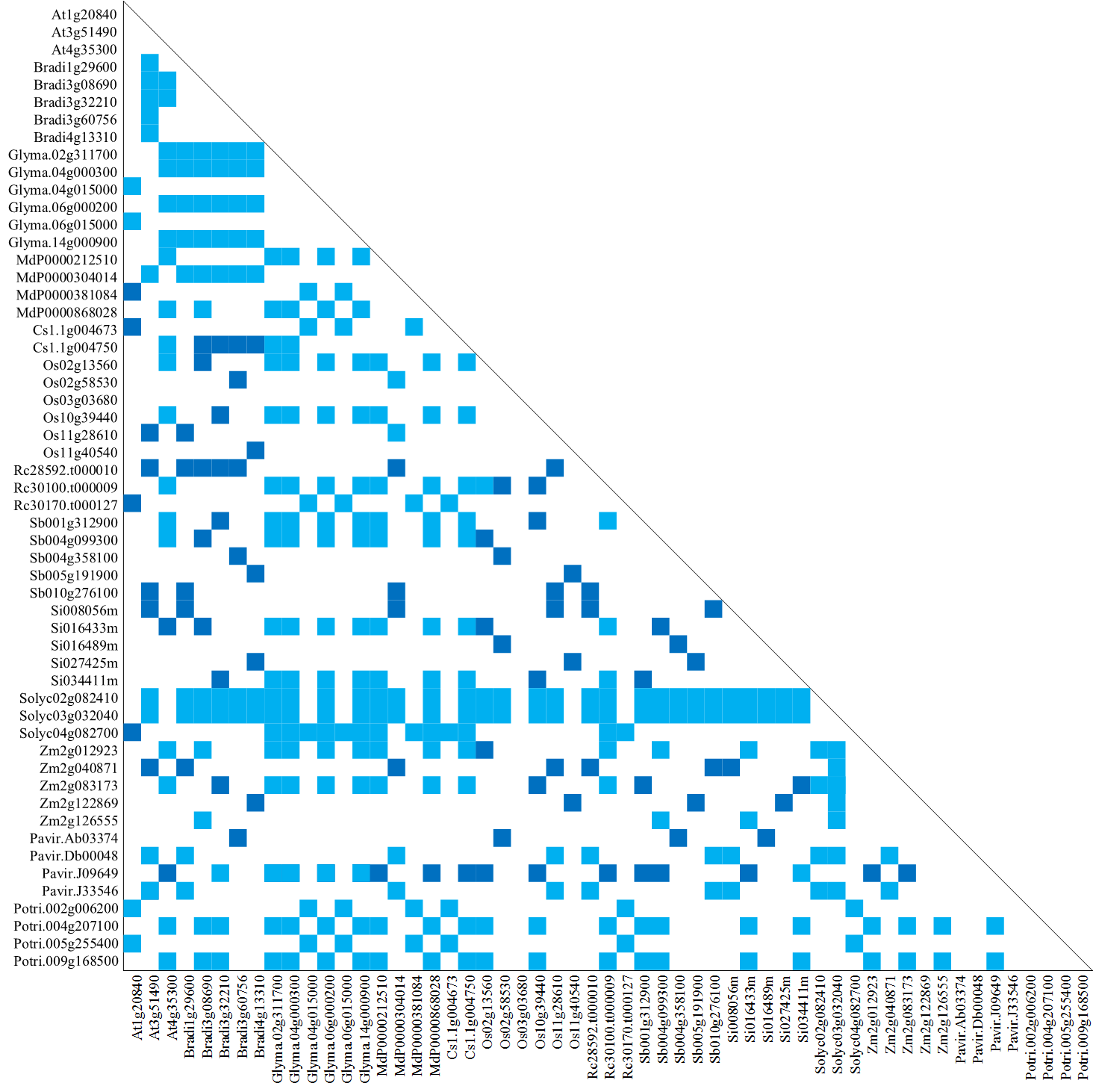


*VGT*


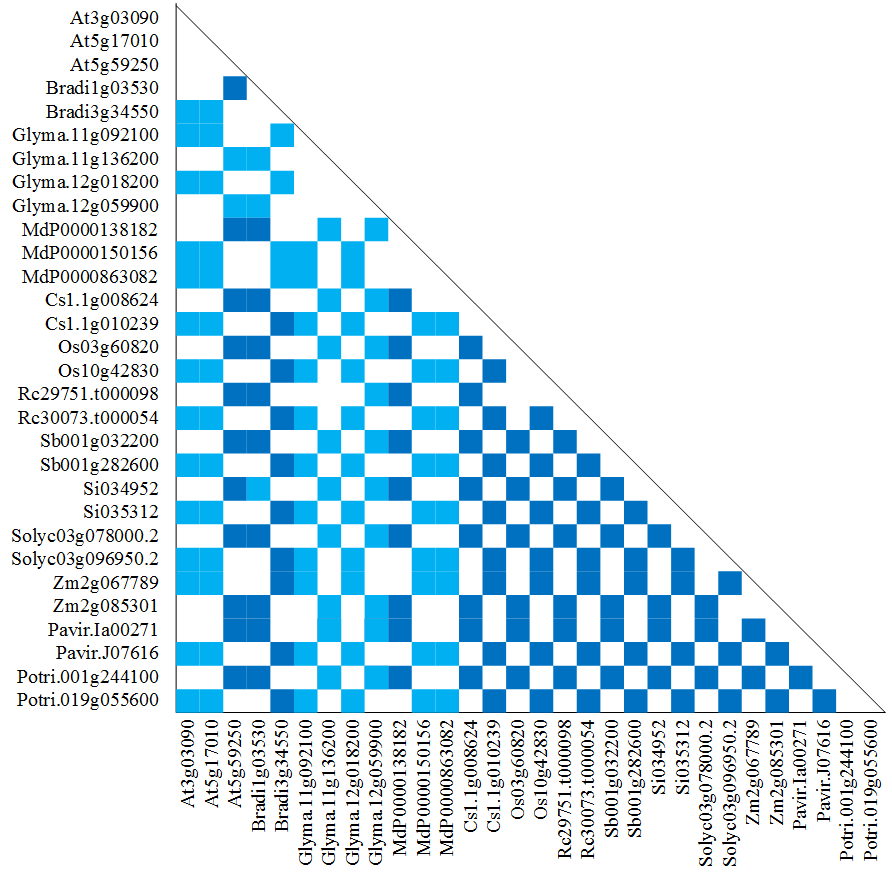


*INT*

*
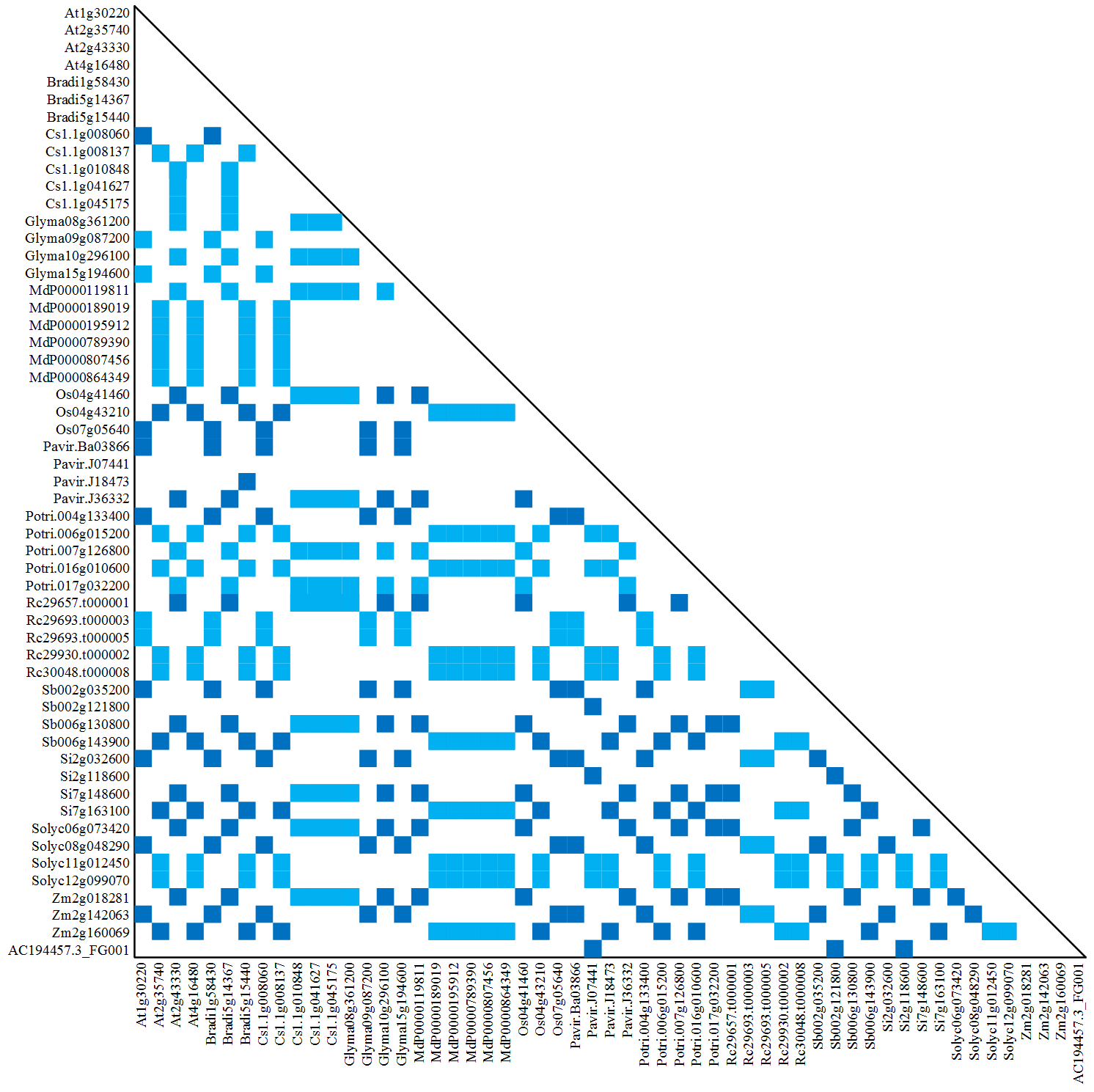
*

*ERD6-like*


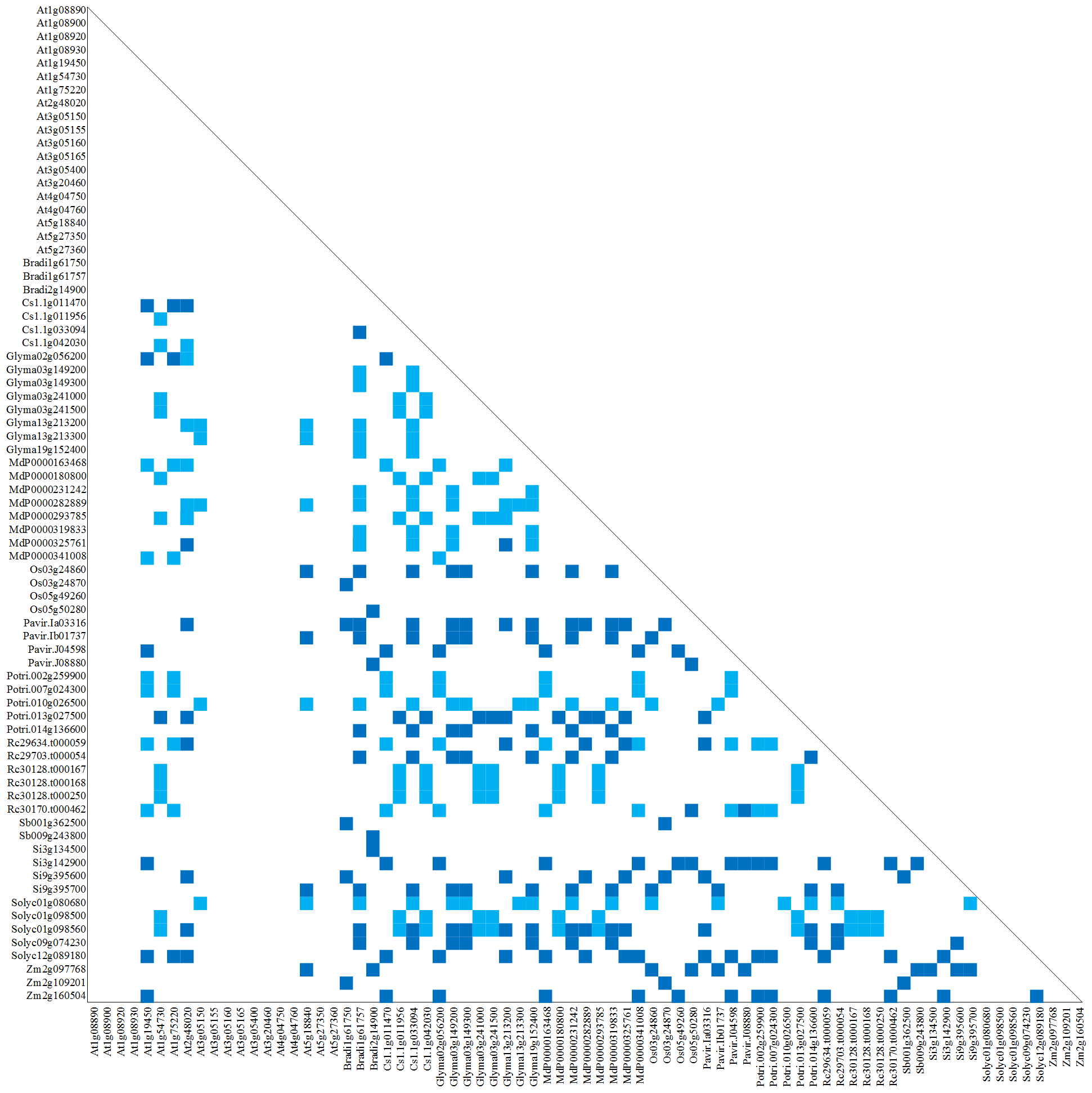


*pGlcT*


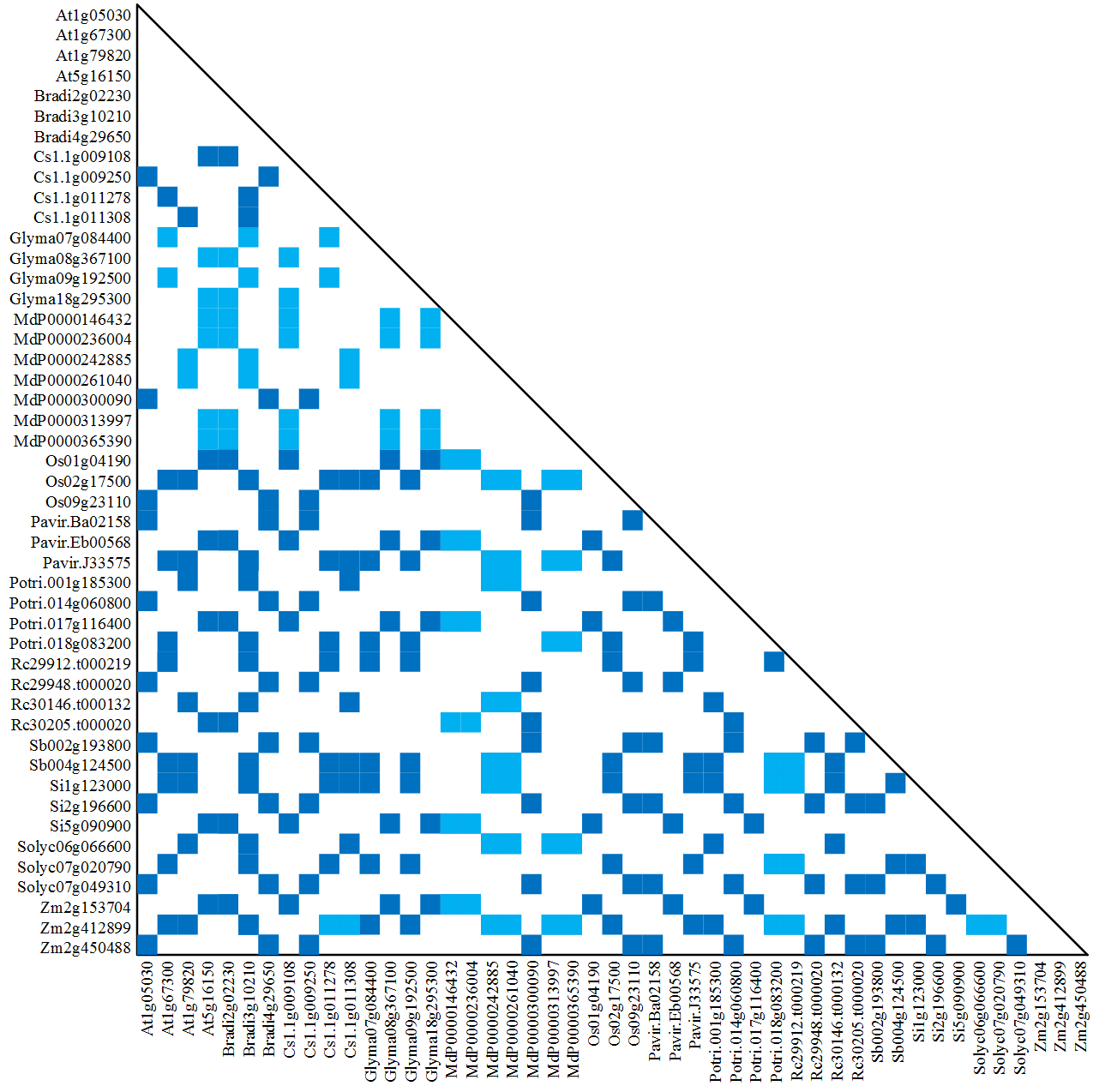


Supplementary Figure S2. Phylogenetic relationship of sequences within each gene family tested in this study. The gene tree was constructed using the neighbor joining method, and bootstrap support above 50% is shown. Red and blue points indicate recent and old gene duplication events, respectively. The dash line indicates gene loss.


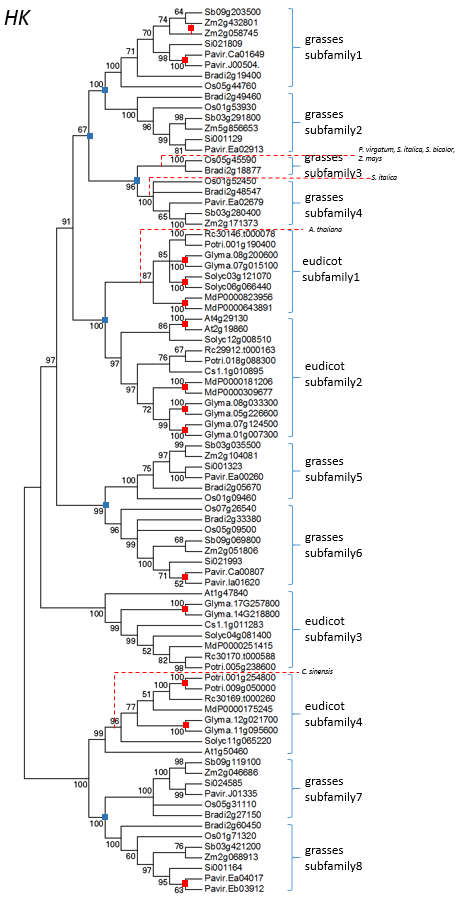


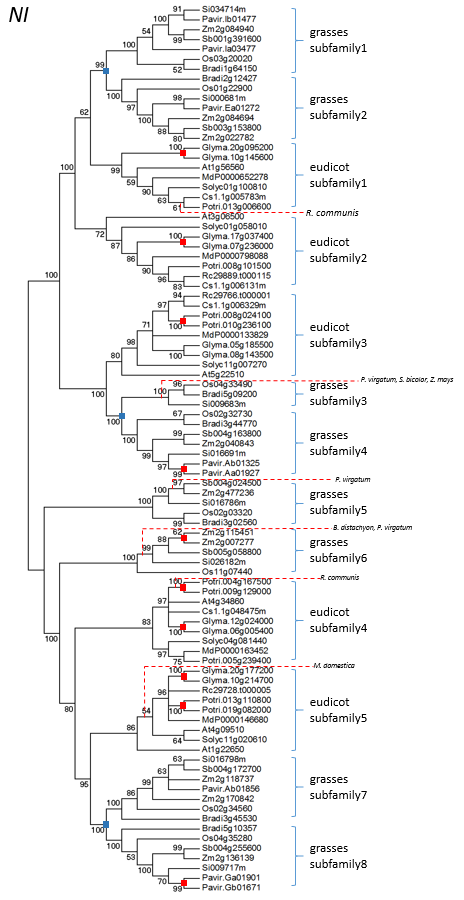


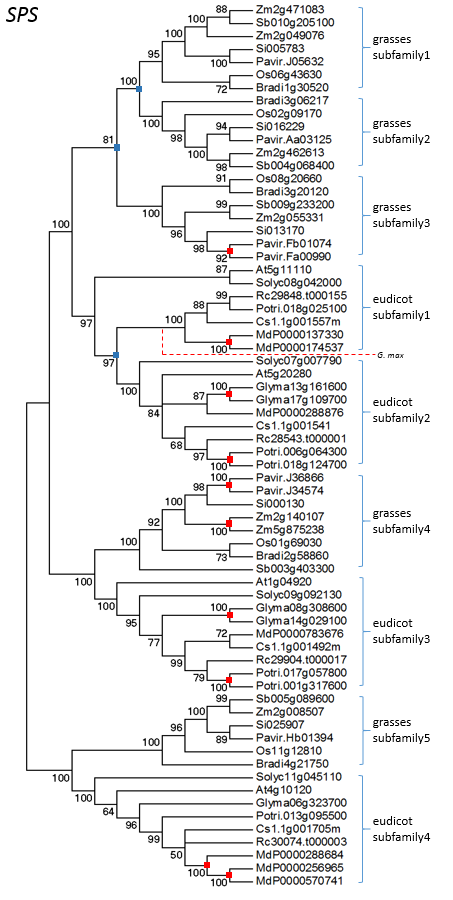


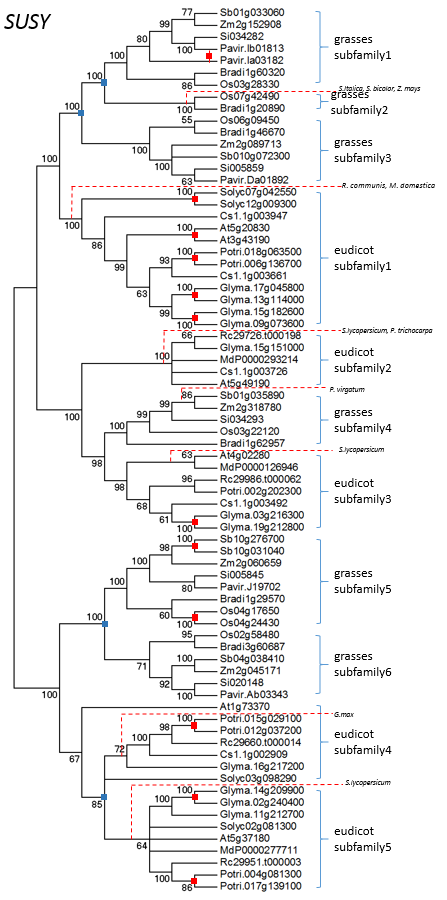


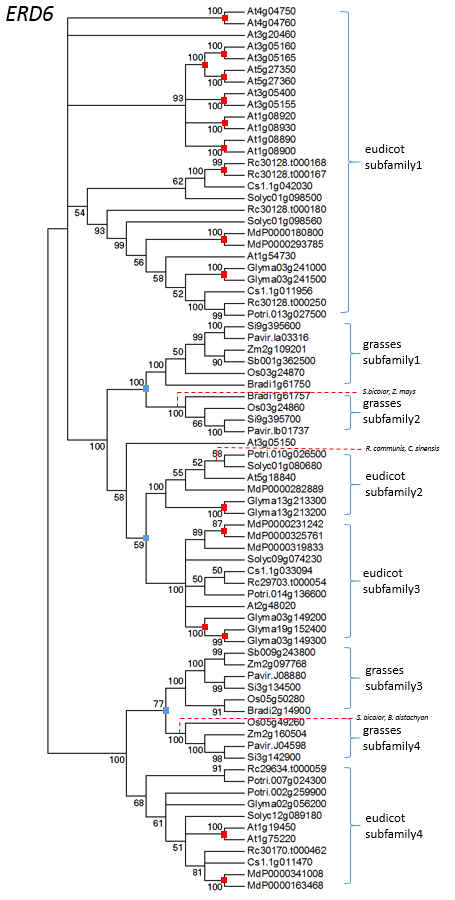


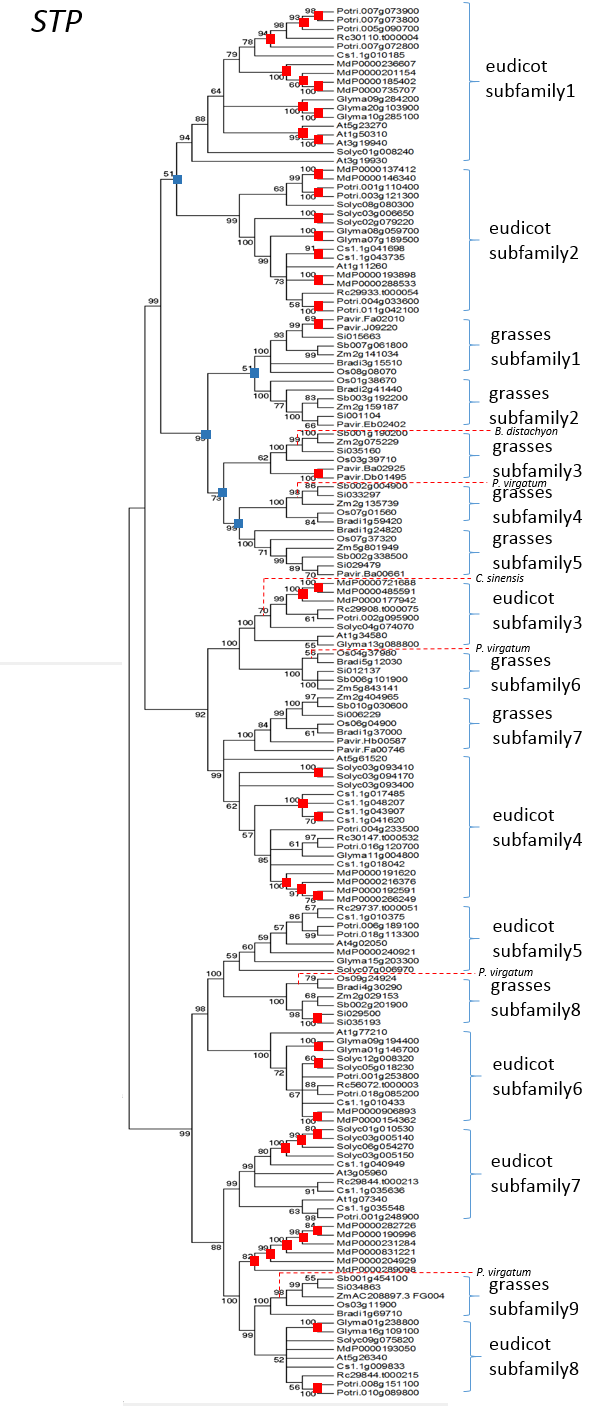


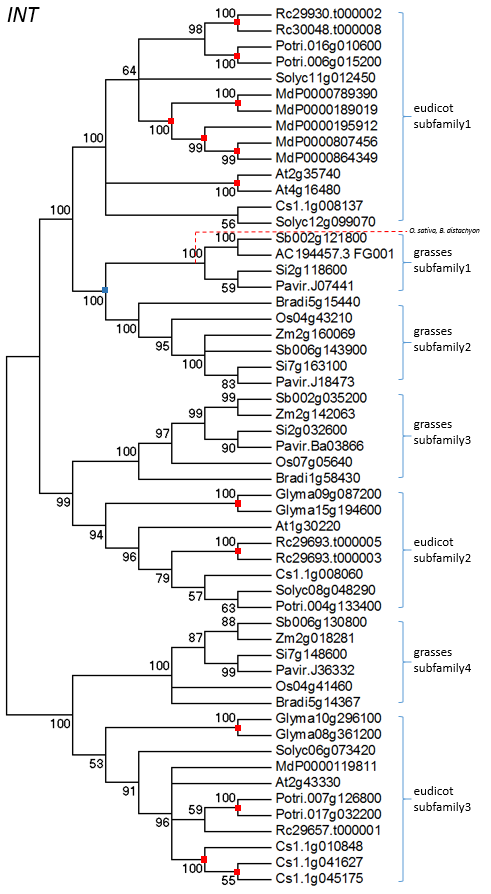


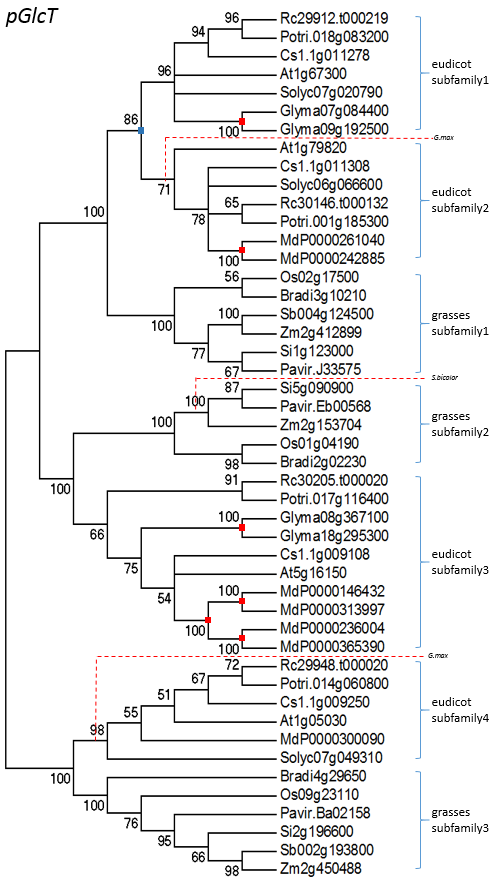


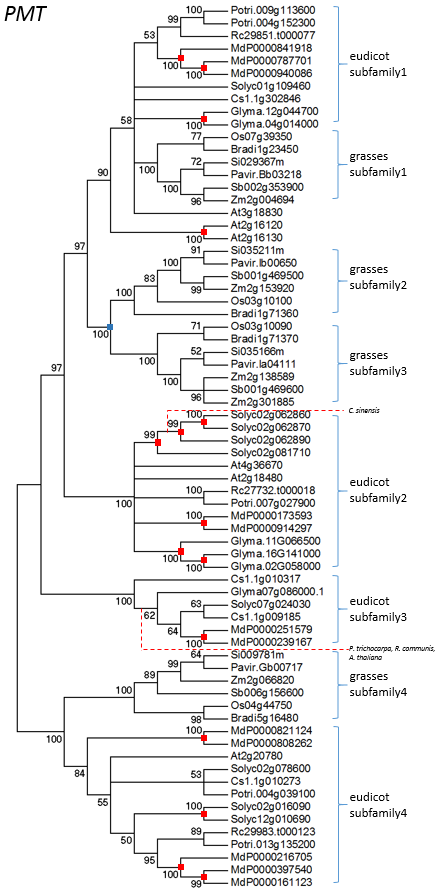


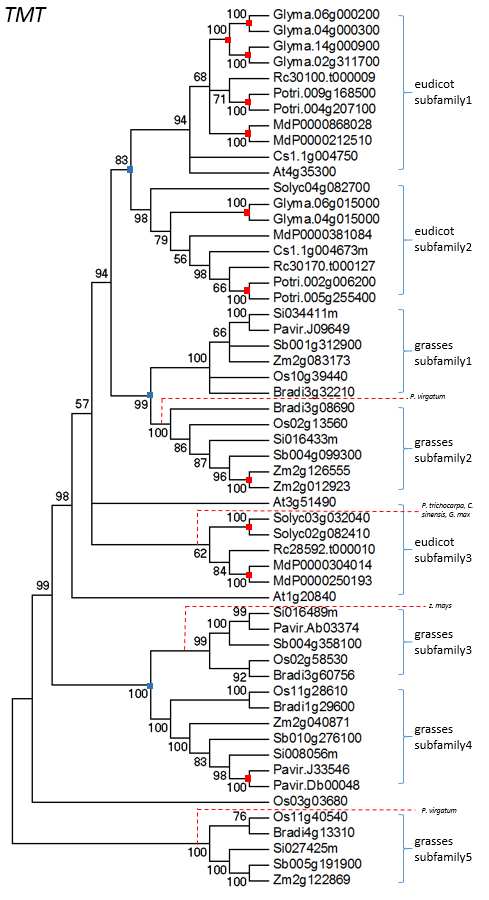


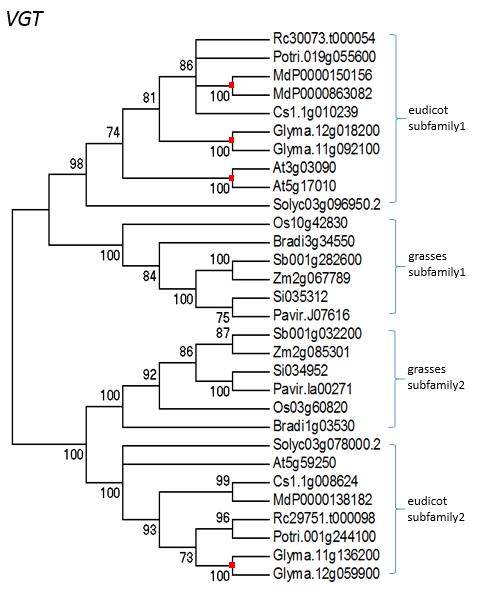

Supplement: Supplementary Information [file srep29153-s1.doc]
